# A 3D taphonomic model of long bone modification by lions in medium-sized ungulate carcasses

**DOI:** 10.1038/s41598-021-84246-1

**Published:** 2021-03-02

**Authors:** Manuel Domínguez-Rodrigo, Agness Gidna, Enrique Baquedano, Lucía Cobo-Sánchez, Rocio Mora, Lloyd A. Courtenay, Diego Gonzalez-Aguilera, Miguel A. Mate-Gonzalez, Diego Prieto-Herráez

**Affiliations:** 1 Institute of Evolution in Africa (IDEA), Alcalá University, Covarrubias 36, 28010 Madrid, Spain; 2 Area of Prehistory (Department History and Philosophy), University of Alcalá, 28801 Alcalá de Henares, Spain; 3 Paleontology Unit, National Museum of Tanzania in Dar Es Salaam, Robert Shaban St., P.O. Box 511, Dar es Salaam, Tanzania; 4 Department of Cartographic and Land Engineering, Higher Polytechnic School of Avila, University of Salamanca, Hornos Caleros 50, 05003 Ávila, Spain; 5 Department of Topographic and Cartography Engineering, Higher Technical School of Engineers in Topography, Geodesy and Cartography, Universidad Politécnica de Madrid, Mercator 2, 28031 Madrid, Spain

**Keywords:** Archaeology, Behavioural ecology

## Abstract

Here, we present the first three-dimensional taphonomic analysis of a carnivore-modified assemblage at the anatomical scale of the appendicular skeleton. A sample of ten carcasses composed of two taxa (zebra and wildebeest) consumed by wild lions in the Tarangire National Park (Tanzania) has been used to determine element-specific lion damage patterns. This study presents a novel software for the 3D spatial documentation of bone surface modifications at the anatomical level. Combined with spatial statistics, the present analysis has been able to conclude that despite variable degrees of competition during carcass consumption, lions generate bilateral patterning consisting of substantial damage of proximal ends of stylopodials and zeugopodials, moderate damage of the distal ends of femora and marginal damage of distal ends of humeri and zeugopodials. Of special interest is, specifically, the patterning of tooth marks on shafts according to element, since these are crucial to determine not only the type of carnivore involved in any given bone assemblage, but also the interaction with other agents (namely, hominins, in the past). Lions leave few tooth marks on mid-shaft sections, mostly concentrated on certain sections and orientations of stylopodials and, to a lesser extent, of the proximal tibia. Redundant occurrence of tooth marks on certain bone sections renders them as crucial to attest lion agency in carcass initial consumption. Indirectly, this can also be used to determine whether hominins ever acquired carcasses at lion kills.

## Introduction

The study of bone surface modifications (BSM) has been crucial to determine agency in the formation of the archaeological and paleontological records. It has also been fundamental in interpretations of hominin-carnivore interactions^1–4^. Additionally, BSM have also been used for determining carnivore type^5–8^, and the potential order of carnivore intervention in assemblage formation and modification^9–12^. Aside from biotic agents and processes, physical modifications on bone surfaces, such as abrasion marks and biochemical modifications provide important information to assess site formation processes and paleoecological conditions of assemblage formation^13^. Once BSM are identified, their anatomical distribution is essential to differentiate among agents and processes participating in the formation, modification and biasing of the paleobiological record. Until now, this has been done comparing BSM frequencies and anatomical distribution according to bone portion, element and animal. A taphotype approach focusing on furrowing damage on distal ends of long bones has also identified carnivore-type specific patterns in which carnivores can potentially be identified^10^. The general interpretation that any given assemblage has been modified by carnivores is of limited value when one tries to address questions like: (a) did those carnivores intervene before or after hominins? (b) Were certain hominin fossil accumulations generated by carnivores and if so, by what type? (c) Were hominins scavenging from felid kills? (d) Did sabertooth felids modify carcasses subsequently consumed by hominins or other carnivores? (with the broad ecological impact that such a behavior may have had in the food chain of those ecosystems); (e) Given the ubiquitous nature of cave deposits, were such assemblages formed by the intervention/interaction of ursids, canids, hyenids and/or felids? (f) What was the degree of the impact of each of these agents if operating on the same assemblage? (g) How does that relate to ecological conditions of food availability, biomass diversity and habitat-specific degrees of competition? (h) Unless we are capable of determining carnivore taxon-specific agency, we will not be able to use taphonomic information for ecological purposes or for reconstructing basic interpretations of hominin-carnivore interactions that are essential for human evolutionary purposes. More simply, until carnivore-specific agency can be done, taphonomists will not be able to provide heuristic interpretations of site formation processes and of site integrity and resolution.

A spatial approach using ArcGIS and the Spatial Analyst Extension was innovated for analyzing BSM distribution and to objectively tally MNE-MNI estimates^14,15^. Recent applications of this software have shown graphic differences in BSM anatomical distribution among different carnivore types^12,16^. Recent evolution of Marean & Abe’s original GIS concept can be found in the use of a more advanced ArcGIS software (ArcMap 10.4) and the elaboration of kernel density maps with Optimized Hot Spot Analysis^16,17^. As some of these authors^17^ acknowledge, for BSM the use of this technique is frequently not adequate, because it requires large sample sizes, while samples under n = 30 are known to produce unreliable results. Its potential use in archaeofaunal assemblages therefore is limited as BSM per element in most cases are relatively few. The advantages in the use of these Geographic Information Systems (GIS) approaches are obvious, but they fall short for providing a proper spatial statistical analysis beyond nearest-neighbour averages and kernel distribution maps. In addition, when graphically displaying their anatomical occurrence, BSM are shown in bidimensional templates, which can vertically be more or less accurate, but certainly less so in the horizontal plane. This is because bidimensional templates cannot reproduce the curved surface of the bone, given that the same mark may occur in more than one view (cranial, caudal, mesial, lateral) at the same time. In addition, one of the biggest criticisms of these methods is that they lack any objective (i.e., statistical) way to carry out inter-assemblage comparison, and interpretations on archaeofaunal assemblages are mostly made using subjective comparisons with experimental controls by the analyst.

As if these were not sufficient cautionary arguments, far from what could be thought, the kernel density maps that these methods are based on come in many types and are determined by the selected smoothing bandwidth. Such a selection is not trivial. The same data, under different bandwidths can result in widely divergent maps. Optimal bandwidths must be selected according to optimization criteria either using plug-in selectors, cross-validation selectors or other methods, such as balloon estimation, Scott’s rule of thumb, Fast Fourier transforms or Diggle’s estimator. Some of these methods make assumptions (i.e., inhomogeneous Poisson process, Cox Process…) that must not be skipped if properly used. In addition, heavy-tailed distributions make bandwidth selection very difficult^18–20^. This impacts the use of these methods in taphonomic work because very frequently, distribution of BSM on bones follow such distribution types. Therefore, it is not surprising to find that bandwidths on the same dataset may differ by more than ten times depending on the method selected. This introduces an element of statistical uncertainty and questions validity of interpretations based on the use of kernels alone. Most taphonomic works using this method to date do not specify bandwidth selection method or justify it in the face of alternatives. This is why kernel methods in taphonomic analysis of BSM are statistically unreliable.

Similar criticisms could be made of more dynamic and complex database interfaces, such as TIPZOO^21^, which for BSM also uses bidimensional templates, with the downside of tallying BSM according to the segment of the bone where the BSM occur, but not to their actual location. Such segments are reduced for epiphyseal portions, but extremely large for shaft portions, making BSM location uncertain and without proper experimental proxies to be interpreted. Despite this criticism, TIPZOO is a great improvement over other interfaces to record zooarchaeological information, translate its databases to R for analysis and then offers very valuable graphic display of descriptive results using QGIS.

Building upon ArcGIS and the Spatial Analyst, we present here a new software tool (Ikhnos), specifically designed for the three-dimensional spatial analysis of BSM. Ikhnos was coded in C++ and using SQL for communication with relational databases. It provides a clear interface that can record field data as well as bone and lithic attributes. The resulting data can be exported directly to R where spatial statistical analyses can be conducted. This enables obtaining three-dimensional taphonomic information and analyzing it to statistically assess the existence of patterns and their nature.

It has been frequently assumed that BSM created by carcass-defleshing agents (i.e., cut marks made by humans and tooth marks imparted by strict carnivores) are unintentional accidents on bone surfaces^22^. Given this purported stochastic nature of BSM, it could be argued that no clear pattern should emerge in tooth mark distribution unless redundancy in the location of tooth marks is conditioned by either carcass morphology, the properties of the dentition of the carnivore agent, its carcass consumption behavior or a combination of these variables. Differentiating among carnivores modifying a faunal assemblage would be an important advance in the reconstruction of the archaeological and paleontological records. This also would have major relevance in paleoanthropological research for determining the type of interactions that hominins had with other carnivores in the past. Recent methodological advances have been made identifying specific carnivore types in archaeofaunal assemblages when analysing BSM through artificial intelligence tools^23^ and other machine learning methods^24,25^. We believe that carnivore-specific patterning in carcass modification could also be feasible if analyzed anatomically in three dimensions. This is the target of the present work. Here, we apply the new Ikhnos software to collect three-dimensional information of medium-sized carcass modifications by lions. The goal is to detect if patterns in carcass modification by lions can be statistically supported. Only under the light of statistically-based patterns can interpretations of felid-hominin interactions be made when analyzing archaeofaunas. The testing hypothesis is that if element-specific patterns exist, they must be reflected bilaterally (against randomness). Initial patterning was visually and graphically documented in earlier studies^12,26^. The present study aims at refining such patterning spatially and statistically. If confirmed, more carnivore-specific patterns ought to be sought to create modern proxies for interpreting archaeofaunal assemblages. Additionally, the present analysis aims at finding the emergent properties of spatially-related sets of marks, understood as point processes, which could identify how intense (i.e., absolute and relative frequencies), clustered, spatially-correlated and independent each mark process created by different agents is. We show that such a meta-analytical information has a great discriminatory power and constitutes a new methodological contribution of the present work.

## Method

### The Ikhnos software

Ikhnos is a new 3D-geospatial software for taphonomic and zooarchaeological analysis. Ikhnos includes some recent innovations in 3D modelling, statistical analysis and geospatial databases for the three-dimensional spatial analysis of BSM. The graphical user interface (GUI) was written using the C++ programming language, while the database management system was developed using MySQL and Qt Creator framework to unify all the different components. Ikhnos is compatible with the most popular geotechnologies for 3D recording such as laser scanning or photogrammetry, allowing for the 3D documentation of taphonomic data on osteological materials. Moreover, Ikhnos is able to incorporate other sources of information such as other essential archaeological information. To digitise and incorporate all this heterogeneous information, a 3D geospatial database was designed which interacts with the main application to store new information or retrieve known data (e.g. bones name, site features, etc.) (Fig. S1).

The Ikhnos GUI is based on a main window divided in two modules (Fig. S2):

1. The first module encloses the database and contains all the associated information that is stored for each item. This part is divided into five sections: (1) site data, (2) bone or lithic item data, (3) intra-site item data, (4) 3D model editing and (5) landmarks.
2. The second module is devoted to the interactive 3D viewer of the each bone’s 3D models using different formats (i.e. point cloud, mesh model, polygonal model).

Regarding the first module, “*site data”* refers to the archaeological site where the item was found. To this end, all the sites stored in the database are shown in a drop-down list. In the case of new sites, the user can manually input this information by introducing the name of the site and the spatial coordinates for each object. The “*Bone or lithic data*” can be completed with information of interest: including fields for taxa, anatomical part, age, laterality and animal size in the case of bones. For lithic materials a field is provided for raw materials. The “*intrasite data*” is the section where the user provides specific information about the item from the site. If chosen the user can leave this information empty, with the exception of the item ID, which is an essential identifier for the managing the database.“3*D editing model*” allows the user to edit the 3D model and to segment the precise regions of interest (Fig. S3).

The final section allows the user to add different spatial landmarks or point to the item by clicking on the precise location of the observation on the 3D model (Fig. S4). Two types of landmarks are available; (1) circular marks consisting in a single point which can be assigned for tooth pits or percussion marks; and (2) linear marks, comprised of multiple points (beginning, middle and end), that can be used to document scores or cut marks (Fig. S4). The coordinates for each of the landmarks are automatically outlined in the textbox and are stored in the database as a new registered spatial point.

Finally, the database can be saved and accessed through the software R for statistical analyses.

### Sample

**Figure 1:**
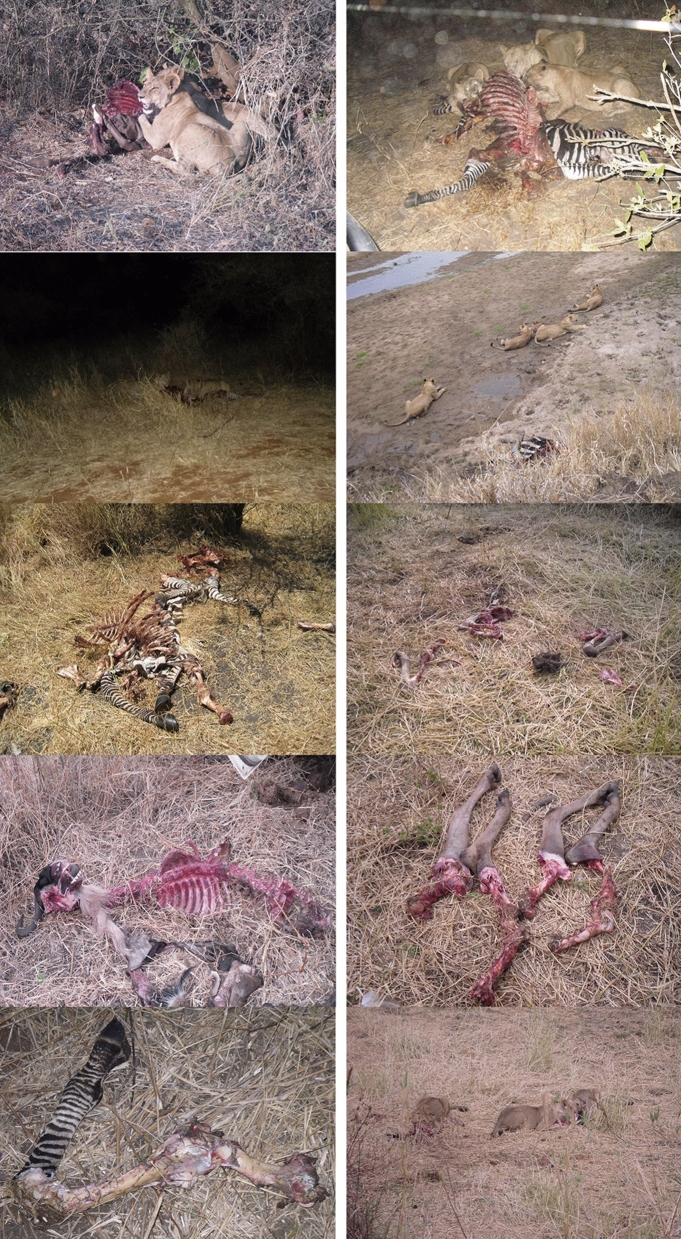
Examples of carcass consumption by lions at Tarangire. Carcasses are arranged by order according to Table 1.

A total of ten carcasses consumed by lions in the Tarangire National Park (north of Tanzania) were selected for analysis. Table 1 shows the distribution of carcasses, including their age, which may have some influence on their modification by lions during consumption, since bones from subadult individuals may be easier to modify by non-durophagous carnivores. It also includes number of feeding individuals, which may also impact how thoroughly carcasses are defleshed and, thus, skeletally modified. These carcasses make part of the larger sample of carcasses studied for three consecutive years by Gidna et al.^27^ and correspond to most of the carcass sample from 2010 (Fig. 1). They were studied because they were easily accessible at the Aguirre-Mturi research Station at Olduvai Gorge and correspond to medium-sized carcasses, which make up the bulk of the lions’ range of prey size. The field study was conducted within the Tarangire Lion Project in 2010 during the months of July–August, corresponding to the overlap of the beginning and the middle dry season. For the study, up to six lion prides were monitored with radio-collars. This enabled monitoring of the six prides simultaneously and early access to their hunts. For the animals used in this study, a minimum of two lions and a maximum of seven lions fed on the carcasses documented. For a detailed description of the study and the complete sample see^27^. In most cases, lions stayed at the kill for a full day and carcasses were abandoned by lions upon complete consumption. Carcasses were treated following the protocols dictated by TANAPA (Tanzanian National Parks). When the pride moved away, bones were collected and cleaned by boiling in a solution of neutral detergent. After drying subaerially, bones were catalogued and stored in metal containers. For the present study, only long bones were used. In none of the carcasses analyzed were metapodials modified in any way. Thus, these elements were excluded from the study.

**Table 1:** Zebra and wildebeest carcasses analyzed in the present study, including the number and type of lion consumers.

Carcass no.	Habitat	Prey	No. of lions	No. of adult	No. of subadult
2	Bush	Wildebeest	7	2	5
4	Bush	Zebra	7	2	5
5	Bush	Zebra	4	3	1
6	Forest	Zebra	4	4	0
7	Forest	Zebra	4	4	0
8	Plain	Wildebeest	7	2	5
9	Forest	Wildebeest	2	2	0
10	Forest	Wildebeest	2	2	0
11	Forest	Zebra	2	0	2
12	Forest	Wildebeest	7	2	5

Bone surfaces were subsequently examined microscopically, aided by the use of hand lenses (10x-20x). Conspicuous and inconspicuous (i.e., confirmed with augmentation) marks were identified and anatomically located. These were then documented using the 3D templates provided by Ikhnos. Only tooth pits and scores were used. No punctures were observed. When furrowing was extensive (i.e., large portions of trabecular tissue were removed) it was not documented (this only was observed in proximal stylopodials). Only when marks appeared associated to the rim of the furrow or the furrow was small enough as to preserve part of at least a single tooth shape, this was introduced into the 3D templates. This decision was based on the difficulty of locating true mid-point coordinates for marks in the case of furrowing. By excluding furrowing, we focused just on tooth-marking, which has never been systematically documented in a descriptive or quantitative manner on long bone shafts before, since previous attempts to document tooth pits and scores were overshadowed by the abundance of furrowing on certain long bone portions, namely ends. Upon completion, the resulting database was exported into R and analyzed as described below.

### Statistical methodology

For statistical analyses, four summary second-order functions were used for exploring how tooth marks were distributed (i.e., clustered, dispersed or randomly) in discrete space portions. The null hypothesis is CSR (Complete Spatial Randomness). CSR, also known as Poisson point process, indicates that points (in this case tooth marks) are locationally independent and display the same intensity (frequency according to spatial area) regardless of location. Ripley’s K function determines the number of points within multiple defined distances^28^. The G cumulative function derives the nearest neighbour distance *G(r)* of a point pattern^29^. The F cumulative function estimates the empty space function F(r) of a point process^30,31^. The three-dimensional pair-correlation function (pcf) estimates point intensity within defined ring ranges. Here, these functions were used creating point envelopes (i.e., proxies for confidence) by implementing a Monte Carlo simulation procedure. A total of 500 simulations were performed. In order to avoid discretization effects, a minimum of 512 values for r from which K3(r), G3(r) and F3(r) are estimated were used. Given the problems with the K-function in inhomogeneous scenarios, an analogue of Besag’s L-function in three dimensions was adopted by using a square-root transformation of the K-function. The three-dimensional K3est, G3est, F3est and pcf3est functions from the “spatstat” library were used in R 3.6.1 (www.r-project.com) for these second-order tests. A distribution above the Poisson process region indicates clustering, and below suggests regular dispersal for the K and G functions. The inverse is true for the F function. For the F function, the theoretical F3(r) value for the uniform CSR process was determined using a discretized distance radius based on the “digital” method.

Patterns of BSM on bilateral elements were compared using two methods. One consisted of using the three-dimension coordinates of the right and left elements to generate a principal component analysis (PCA). The results were plotted using 95% confidence ellipses of data points for each element. The overlap or distance in between ellipses determined the similarity or divergence in the patterns documented on each element.

The second method was based on wavelet coherence analysis^32^. Longitudinal data, especially those in time-series format, may present redundant patterns and localized outliers, which are well identified through spectral and wavelet analyses. Spectral analysis carries out a distribution of any given sample’s time-series variance over frequency. It is one of the best methods to detect periodic signals and patterns difficult to detect due to fluctuating noise. It can also be used for detecting non-normal fluctuations in the form of peaks in the longitudinal series spectrum. The use of sine waves (based on cycles and harmonic series) for smoothing spectra unveils fundamental frequencies and exceptional frequencies. In traditional spectral analyses, Fourier transform algorithms are used to detect a Fourier series in a longitudinal sequence. Wavelet analysis has come as a new generation of sinusoidal waves to detect patterns in spectral analysis. Wavelets are smaller waves created through modifications of the Fourier basic functions. They have been defined as “objects that oscillate and decay fast, and “hence” are little^33^. Some of the important characteristics of wavelets are that they are efficient feature extractors and, therefore, that they also succeed at extracting underlying structures. They are efficient also in computation time being faster than the Fourier transform. We incorporated wavelets in our analysis because they are great tools for multiscale analysis of sequences expressed as vectors.

Wavelet coherence analysis (WCA) (as a bivariate wavelet tool) is used to detect any relationship or pattern between two signals. A wavelet coherence plot can be used to detect if the two signals are highly correlated (usually represented in red) or not correlated (usually represented in blue). Wavelet coherence analysis can be used for spatial analysis^34^. Here, we adopt the approach of analyzing long bone length spatially as a continuous metric sequence with equal opportunity of being impacted by carnivores (null hypothesis). For this purpose, each long bone was divided into a series of small bins (see Table 2) and the number of BSM falling in each of these portions was tallied. Only the central point of each mark was taken as a reference. The vectorized information was analyzed through WCA using the “wtc” function of the “biwavelet” R library. The resulting coherence plot displayed longitudinal information on the horizontal axis referring to inter-epiphyseal distance (distal on the left and proximal on the right). The vertical axis shows the scale. Here, the scale is inversely proportional to the frequency (the lower the frequency, the higher the scale). Non-uniform patterns resulting from localized concentration of BSM were taken as defining the alternative hypothesis. Given the small distance between the mesio-lateral and cranio-caudal surfaces, no WCA was performed on those axes. For all the axes used, histograms with density distributions were used to document the frequency of BSM along the three-dimensional surface of the bone.

**Table 2:** Average values for observed data in each second-order function (K,G,F and pcf) and nearest-neighbour density (nnd) and intensity.

	K_obs	F_obs	G_obs	pcf_obs	nnd	Intensity
lHum	1114748	0.855585	0.7011798	1.356317	9.898941	56
rHum	967047	0.8535749	0.9122934	1.50223	9.237289	61
lFem	1845998	0.8491423	0.9156585	6.00765	10.77651	46
rFem	1784145	0.8656865	0.9355373	6.342219	8.009646	97
lRad	2986224	0.7896682	0.9540846	4.728153	8.367454	31
rRad	1136433	0.7806659	0.9162847	4.56088	8.964272	22
lTib	1173525	0.8209044	0.9451497	1.426124	14.07071	19
rTib	492396.3	0.7658673	0.8836768	1.581524	13.13529	23

Finally, to compare the patterning of tooth mark modifications on the whole appendicular skeleton, a selection of variables was made. These were: the mean of the observed values of the K-function, the G-function, the F-function, and the pair-correlation function, the average of the nearest neighbour distance and the global intensity. These variables were analysed via a hierarchical cluster analysis. This was done using an Euclidean distance matrix of the original values of the variables. For the analysis, the “average” method was selected.

## Results

### Humerus

**Figure 2:**
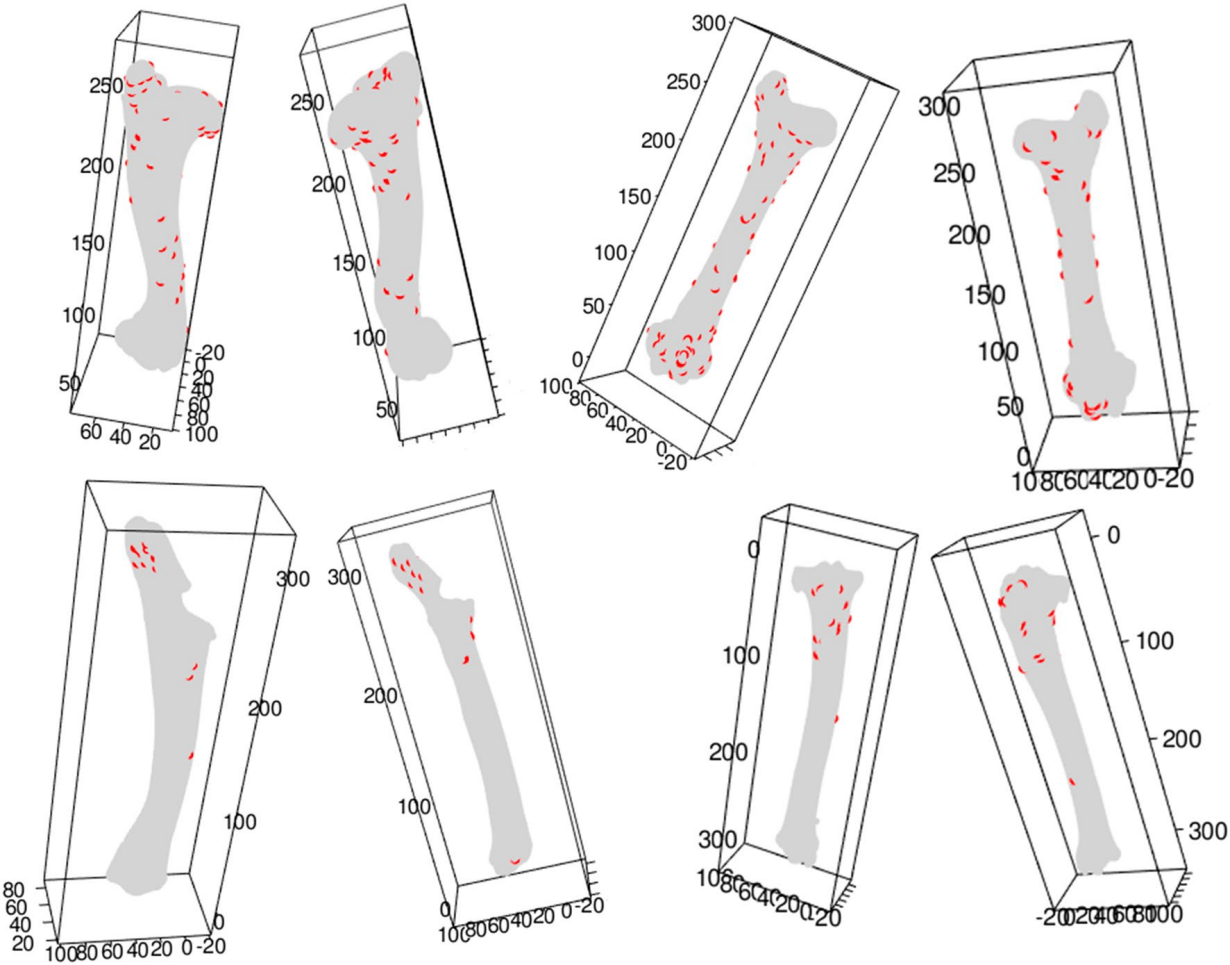
Examples of three-dimensional tooth mark distribution from the lion-consumed carcass sample on each of the four long bones. Distribution of marks is shown on bilateral representation.

Left humeri display a non-stationary (i.e., spatially variable in intensity) distribution of tooth marks, with inhomogeneous intensity and with a clustering trend, especially on the proximal end. Tooth marks cluster on the proximal epiphyses, both on the tubercles as well as the articular surface area and proximal metadiaphyses. They also occur in the vicinity of the deltoid crest. Shafts present more abundant modifications on the caudal and medial sides. The cranial and lateral distal shafts show very few tooth marks in comparison. This distribution shows a connection between tooth mark occurrence and areas of muscle and ligament insertions. Tooth marks were probably created during defleshing and limb detachment from the trunk. They are most abundant on the neck junction between the articular head and the proximal metadiaphysis (Fig. 2).

**Figure 3:**
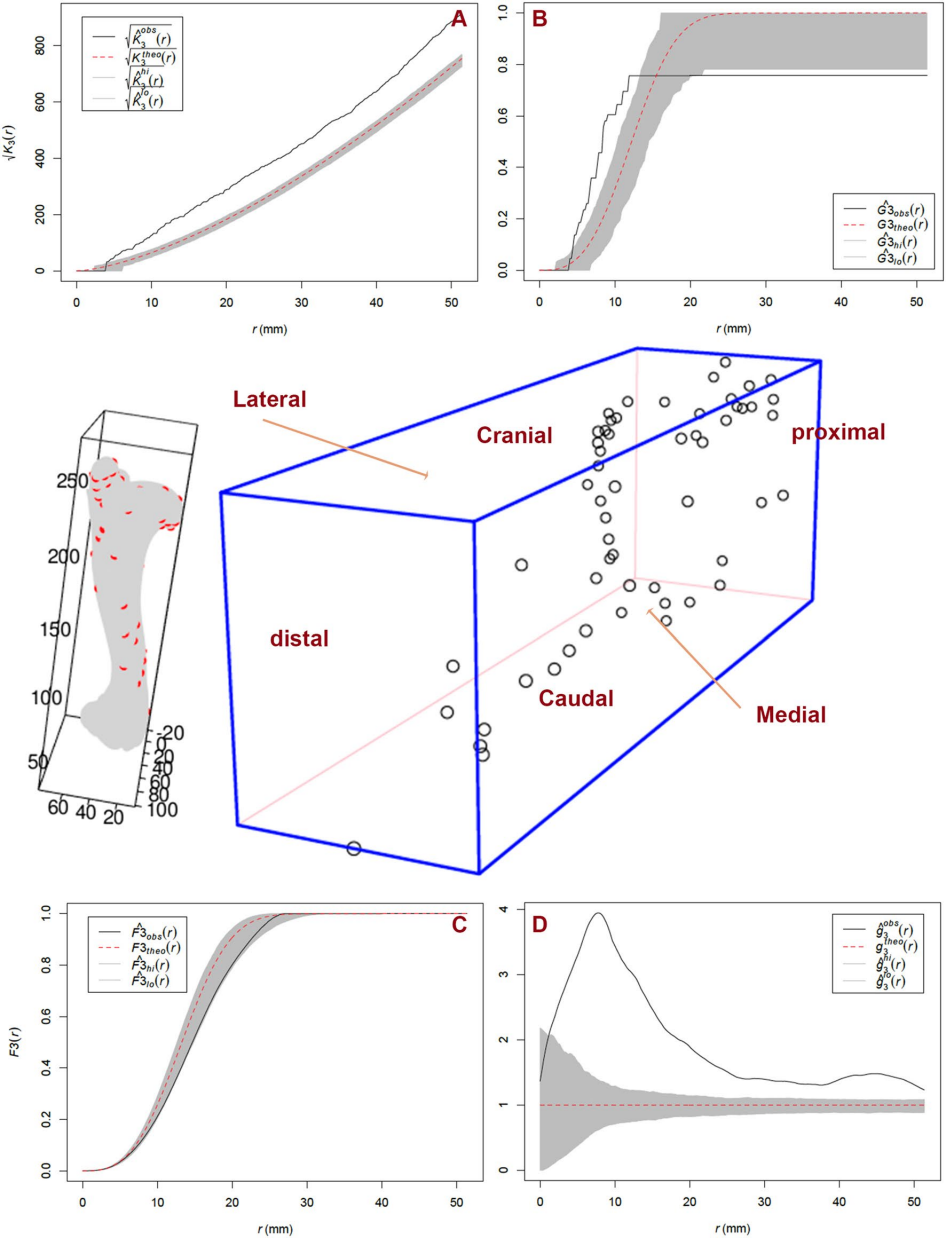
Three-dimensional plot of the distribution of tooth marks on the left humerus. (**A**) K-function plot. (**B**) G near-neighbour function plot. (**C**) F empty space function. (**C**) Pair-correlation function. The F function suggests a pattern non-differentiable from CSR. The other three functions suggest a mild clustering trend in short distances. Key to (**A,B,D**) Dotted red line shows the Poisson Complete Spatial Random (CSR) process and the gray band shows its confidence envelope. Black line shows the point process of the target sample (here, tooth marks on humerus). When above the CSR Poisson process, it indicates a clustering trend. When below, it indicates a regular scattering trend. The interpretation is reverse for (**C**) (F empty space function). Same interpretation applies to equivalent figures in the Supplementary Information.

Both, the K function and the pair-correlation function indicate an overall trend of clustering. This is nuanced by the other functions. The near-neighbour G function shows a slight clustering trend in short distances and a general asymptotic trend of dispersal in longer distances. The empty-space F function suggests a trend towards clustering within an overall CSR pattern (Fig. 3).

Right humeri show a similar tendency of mark clustering around the neck under the articular surface with both tubercles impacted. Marks on the medial shaft are slightly more abundant than on the lateral shaft, while the latter shows higher concentrations around the deltoid crest. Interestingly, marks on the shaft cluster on the proximal and distal portions and the mid-shaft is mostly devoid of marks, regardless of orientation. The cranial side, especially the shaft, is again the least impacted by lions. All the functions show a moderate tendency to clustering; so much so in the K and pair-correlation functions because the latter is a modified version of the former (by using rings within the distance radius). The G function shows a very slight clustering trend in short distances, which in the F function is barely outside the CSR envelope (Fig. S4).

In sum, the slight clustering trends in both sides indicate a redundant pattern of tooth mark location. This shows that mark distribution in humeri is not random, since it is repeated across all the carcasses studied.

### Femur

Left femora do not show a more widespread distribution of tooth marks than documented in both humeri. Most tooth marks also occur on the proximal half of the element. Most distal tooth marks appear concentrated on the epiphyses. They occur mostly on the medial condyle (on its medial facet) and on the medial portion of the trochlea. Marks on the proximal end occur on the trochanters and also on the spiral line of the neck. The lateral sides of the shaft are the least modified, followed by the caudal distal shaft. Tooth marks on the caudal shaft occur on both sides of the line aspera. As was the case with the humerus, a large portion of marks appear at or near muscle insertion areas. All the functions show a slight clustering trend in short distances and a CSR pattern in longer distances (Fig S5).

Right femora appear substantially more toothmarked than the left ones. Again, the proximal and distal ends exhibit the highest amount of marks. Both trochanters and the proximal metadiaphysis contain large numbers of modifications. Marks on the distal epiphysis occur both on the medial facet of the throclea and on both condyles. Marks on the caudal shaft, along the linea aspera, are more abundant than on the cranial shaft. All functions coincide in finding a moderate clustering trend, which indicates that BSM are not following a CSR pattern (Fig. S6).

As was the case for humeri, the non-random and moderately clustered pattern shows that there are locations, mostly coinciding with tendon and muscle insertions, that are more prone to be impacted by lions during carcass consumption than others.

### Radius-ulna

Radii from carcasses consumed by lions are generally left unmodified^12,26,27^. Most of the damage concentrates on the olecranon of the ulna (Fig. S7). Only a few tooth marks have been documented scattered on the proximal metadiaphysis, some under the articular facet of the lateral epiphysis. The rest occur mostly in the form of isolated marks, without any specific preference for clustering or side. The left radius shows this distribution. Marks outside the ulna are very few and occur on the cranial and lateral sides of the proximal metadiaphysis, in proximity to the articular facet. Scattered marks can be observed on the distal end. In contrast with the stylopodials, the left radius-ulna shows more intense clustering of tooth marks, as denoted by the K,G, F and pair-correlation functions (Fig. S4). This may be the effect of the intense damage on the olecranon.

The right radius appears also very slightly toothmarked, despite the large number of carcasses involved. Most tooth marks concentrate on the ulnar olecranon, with very few scattered along the ulnar shaft and even less so on the radial shaft. The few tooth marks documented on the shaft appear on the uppermost cranial shaft and a couple on the lower caudal shaft. As was the case with the left radius, the second-order functions indicate a clear clustering of tooth marks in slightly longer distances than documented in the stylopods (Fig. S8).

In sum, marks in radii are few and mostly clustered on the ulna. Those on the radial shaft are scattered but also seem to be in connection with damage on the proximal end imparted during defleshing by lions.

### Tibia

The left tibia shows a concentration of tooth marks on the proximal end, more specifically, on the epiphysis and, especially, on the crest. Marks on the shaft are not common and they cluster mostly on the lateral and medial sides and on the lateral portion of the caudal side. Marks in the lower half of the shaft are uncommon regardless of orientation (Fig. S9). This element exhibits the lowest frequency of marks of the whole long bone set. The second-order functions indicate a very minor clustering trend in short distances, probably caused by redundancy in damage in the proximal portion of the element, but most of the shaft, where the few scattered marks occur, seems very similar to a Poisson process. This suggests that damage to the tibia (with the exception of the crest and proximal end) is more stochastic than on the other elements.

Right tibiae are only slightly more toothmarked than the left tibiae. Given its overall greater length than other long bones, its low toothmarking frequencies renders them the least impacted elements in number of tooth marks. Most marks cluster on the proximal end, more specifically, on the tibial crest. The lateral side is more damaged than the other sides. In the whole collection, only one tooth mark was found in the distal half of the shaft (Fig. 2). Again, most of the damage on the caudal side was concentrated on the proximal lateral side, coinciding with the more intensive damage on the lateral portion of the cranial side. The second-order functions suggest also a very minor clustering trend, slightly more marked than on left tibiae, probably because all tooth marks documented concentrate on the proximal half of the element (Fig. S10).

In sum, tibiae show some of the least intense point processes resulting from toothmarking by lions on long bones. Marks occurring on the shaft are usually isolated and more random than on other elements, where they are more spatially recurrent.

### Bilateral element comparison

**Figure 4:**
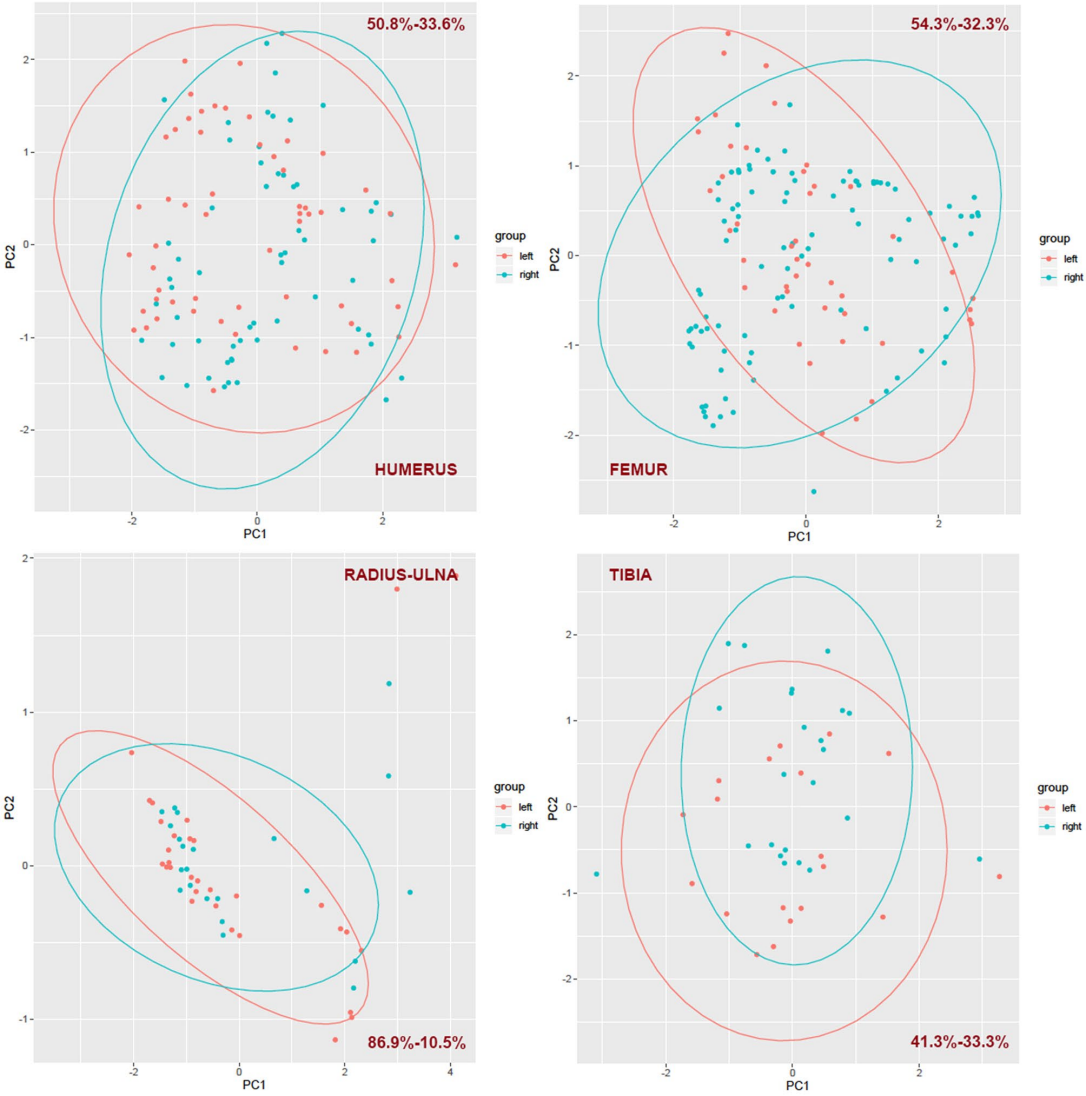
Principal component analysis (PCA) of each of the four long bones (humerus, femur, radius-ulna and tibia) according to side (left–right) showing point distribution according to components generated by compressing the three-dimensional coordinates. A 95% confidence ellipse per side shows variation and similarity of toothmark patterns in each of the bones. Percentages shown are for the first and second component respectively.

**Figure 5:**
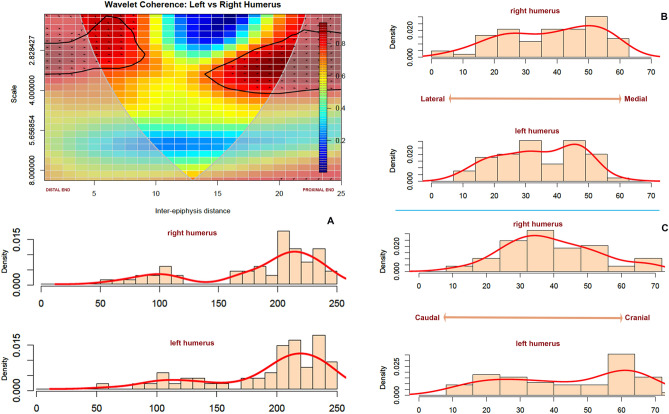
Bivariate wavelet coherence plot showing the correlation of most tooth mark damage on the proximal and distal sections of left and right humeri in low frequencies. Arrows indicate that in these two high-correlation areas, both humeral sides are in phase (i.e., the covary together in the same direction). In the distal area, the right humerus is leading (arrows pointing to the right-down or left-up) and in the proximal area, the left humerus leads (arrows pointing to the right-up or left-down). Binning of histograms is described in Table 3. (**A**) frequency of marks from distal end (left) to proximal (right) end; (**B**) frequency of marks from lateral (left) to medial (right), and, (**C**) frequency of marks on caudal (left) to cranial (right).

Left and right humeri display a similar pattern in the location of most damage as the three-dimensional coordinates of the PCA show (Fig. 4). This is reinforced by the bivariate wavelet analysis, which shows that both sides of humeri show a strong correlation (> 0.8) in the location of most tooth marks in specific locations (Fig. 5). Both humeri display high frequencies of tooth marks (remember, the lower the frequency, the higher the scale) and a clear clustering on the proximal epiphysis and proximal metadiaphysis, as well as on the distal shaft. Most of the mid-shaft shows almost no tooth marks and when they do, they occur in very low frequencies. High frequency marks have only been documented on the proximal epiphyseal portion (Fig. 5). The frequency distribution also shows that the medial and caudal sides bear more marks than the lateral and cranial sides. Most cranial marks are concentrated in the articular surface, tubercles and metadiaphyseal portion of the proximal end.

**Table 3:** Binning of histograms according to bone length.

	x	y	z
Humerus	25 (0–250)	10	10
Femur	28 (0–280)	10	10
Radius-ulna	30 (0–300)	10	10
Tibia	35 (0–350)	10	10

**Figure 6:**
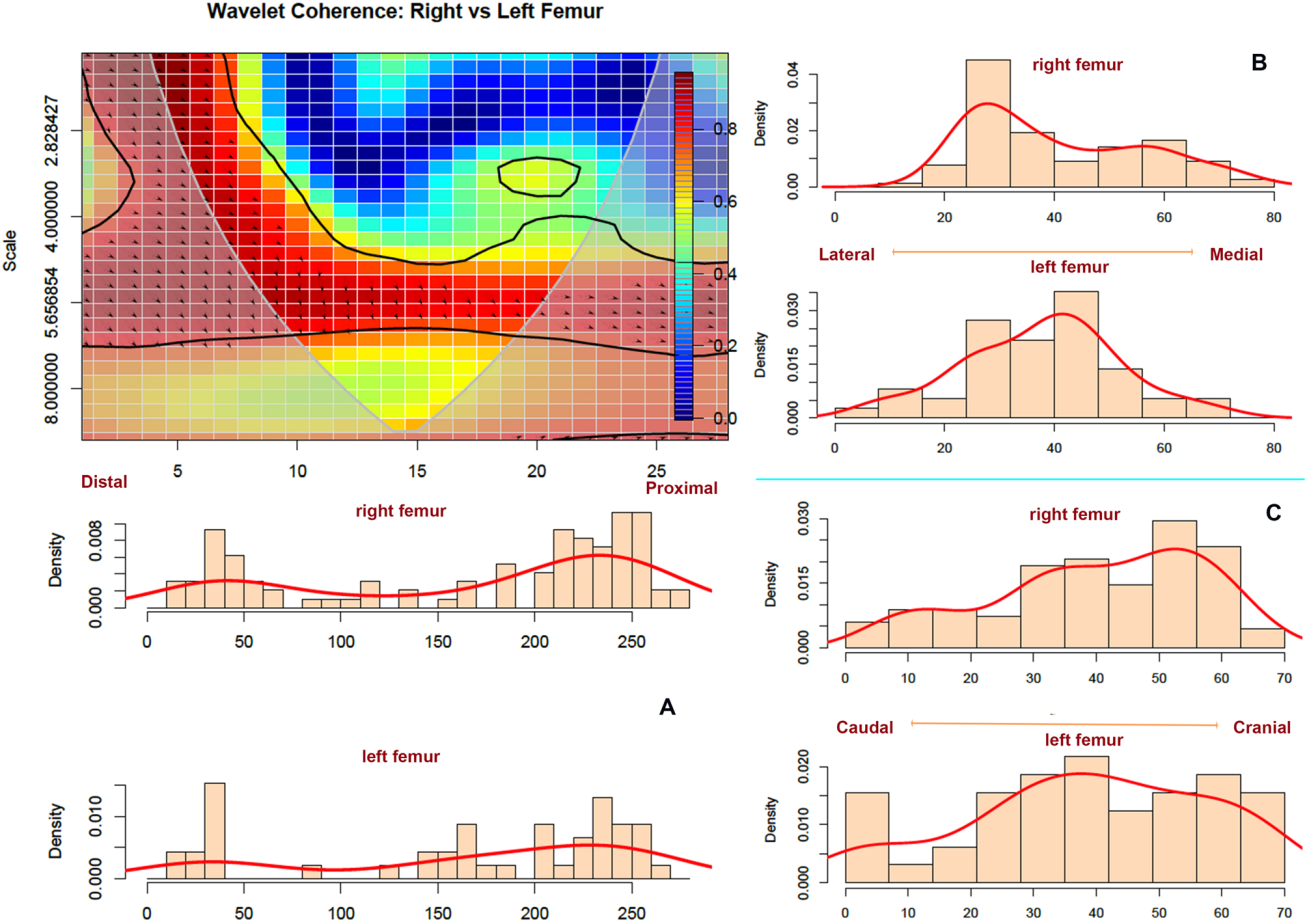
Bivariate wavelet coherence plot showing the correlation of tooth mark damage on the proximal and distal sections of left and right femora in moderate frequencies. Arrows indicate that in these two high-correlation areas, both femoral sides are in phase (i.e., the covary together in the same direction). The right femur is leading (arrows pointing to the right-down or left-up). Binning of histograms is described in Table 3. (**A**) frequency of marks from distal end (left) to proximal (right) end; (**B**) frequency of marks from lateral (left) to medial (right), and, (**C**) frequency of marks on caudal (left) to cranial (right).

Left and right femora also display a similar toothmarking pattern (Fig. 4). Both 95% confidence PCA ellipses overlap in most of their areas. The wavelet coherence analysis shows that both sides display a high correlation (> 0.8) in toothmarking on proximal and distal ends as well as on the shaft when the frequency of marks is low or moderate. Most marks occur on the proximal portion of the element, with a higher impact on the cranial side and more medial for the left femur and more lateral for the right one (Fig. 6). Femoral mid-shafts, thus, appear more highly toothmarked than humeral shafts. Interestingly, the wavelet analysis also shows that when modifications are abundant, there is correspondence between left and right sides only at the distal end. This seems to respond to bone and muscle insertions and ways in which lions deflesh carcasses at this part of the limb. A moderate correlation (> 0.6) between both sides of the element can be found at the level of the proximal articular neck (metadiaphysis) and surrounding the trochanter section (see yellow islands at the level of the 20th-23rd bins in Fig. 6).

**Figure 7:**
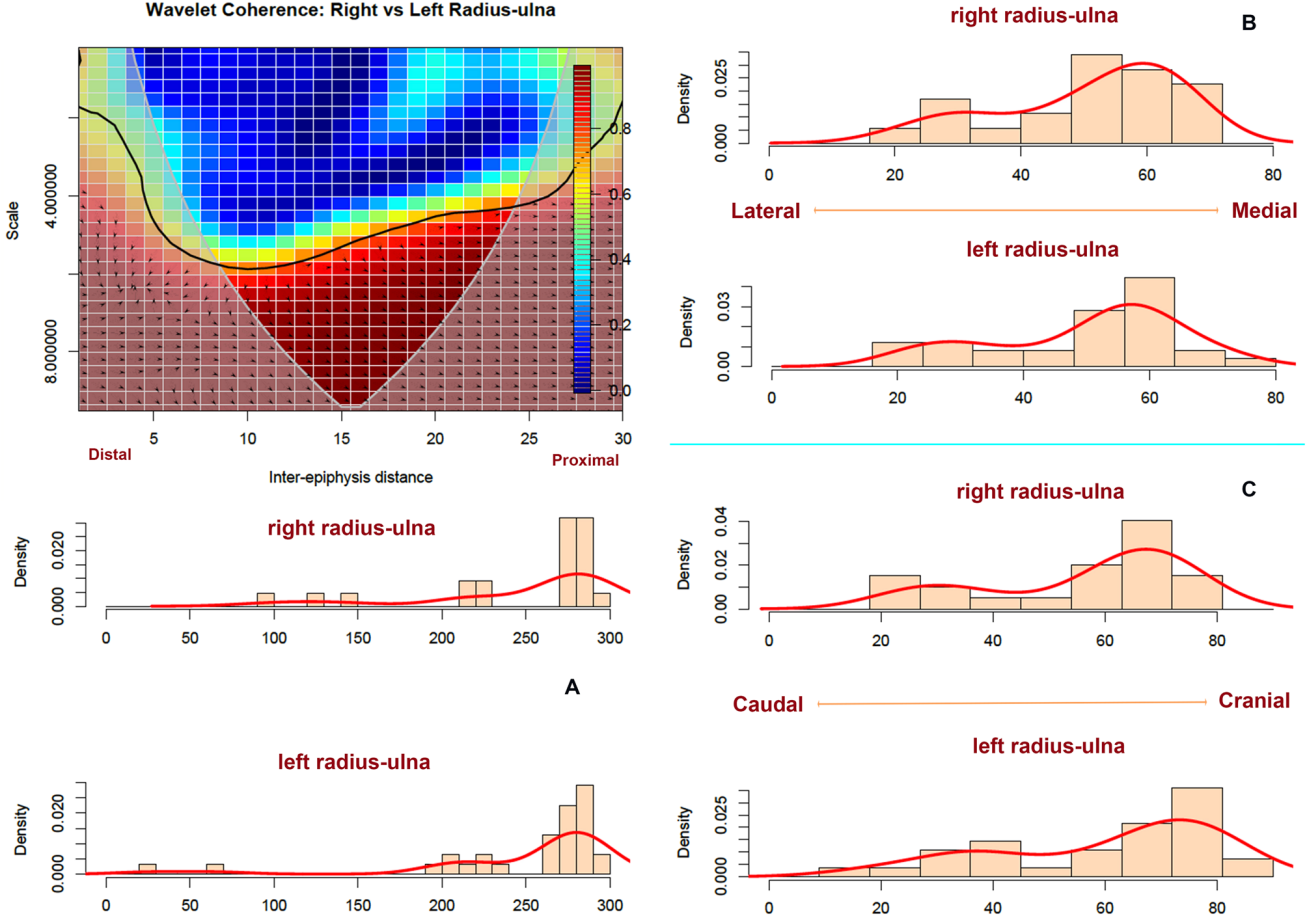
Bivariate wavelet coherence plot showing the correlation of tooth mark damage on the proximal and distal sections of left and right radius-ulna in moderate to high frequencies. Arrows indicate that in these two high-correlation areas, both femoral sides are in phase (i.e., the covary together in the same direction). The right radius-ulna is leading (arrows pointing to the right-down or left-up). Binning of histograms is described in Table 3. (**A**) frequency of marks from distal end (left) to proximal (right) end; (**B**) frequency of marks from lateral (left) to medial (right), and, (**C**) frequency of marks on caudal(left) to cranial (right).

As was the case of the upper limb bones, radii-ulnae also exhibit a localized tooth mark pattern. The 95% confidence PCA ellipses overlap for both sides is more intense even than with the stylopodials. The only points falling outside the confidence ellipse are those that appear in the form of single marks and are caused stochastically. The wavelet coherence analysis indicates a strong pattern between both sides, with marks clustering in the proximal epiphysis and strong correlation in the exhibition of low-impact modifications (i.e., few isolates marks) in most of the shaft (high scale = low frequency). There is a high frequency of modifications on the proximal end (see black line sloping upwards in Fig. 7), which decreases as we go down the shaft. The low frequency is maintained throughout the length of the shaft. Only because a few more marks have been documented on the distal and proximal ends, do we see a lower scale (i.e., higher frequency) at the beginning and end of the plot. The high correlation spread along the element shaft indicates that both the right and left radii-ulnae display virtually the same modification pattern.

**Figure 8:**
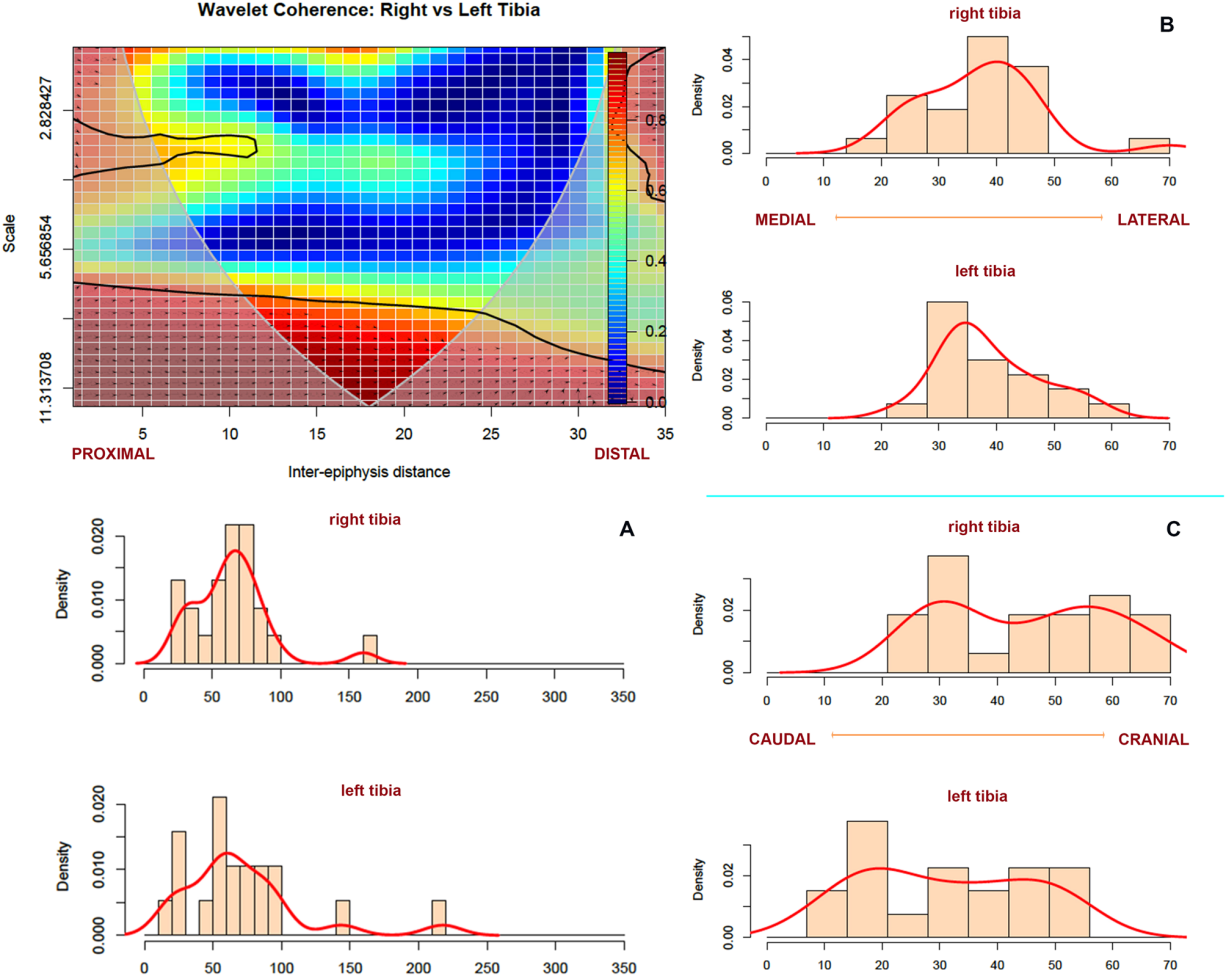
Bivariate wavelet coherence plot showing the correlation of tooth mark damage on the proximal and distal sections of left and right tibiae in moderate to high frequencies. Notice different location of proximal and distal ends compared to the other elements. Arrows indicate that in these two high-correlation areas, both tibial sides are in phase (i.e., the covary together in the same direction). Binning of histograms is described in Table 3. (**A**) frequency of marks from distal end (right) to proximal (left) end; (**B**) frequency of marks from lateral (right) to medial (left), and, (**C**) frequency of marks on caudal(left) and cranial (right).

Tibiae also show similar tooth-marking patterns when comparing right and left sides of the skeleton. A PCA shows that a 95% confidence ellipse of samples from both sides overlap in most of their areas (Fig. 4). However, it should be remarked that there is more coordinate variation (i.e., variation in distribution) of tooth marks in tibiae compared to the other long bones. The reason may be double. On the one hand, the tibia exhibits the longest length dimensions of the appendicular skeleton. On the other side, the occurrence of tooth marks outside the area surrounding the tibial crest is commonly in the form of isolated marks that are more prone to occur randomly during defleshing because no muscle insertions occur on the cranial aspect of the element. Only in the proximal caudal side are tooth marks more prone to cluster because of the muscle insertions on that side. A wavelet coherence analysis shows that tibiae show a low density of modifications, similar to radii-ulnae but over a more widespread area. This creates a situation of high correlation between the left and right sides in the location of the few scattered marks (Fig. 8). The correlation is also similar in the proximal and distal ends when modifications are more clustered. Overall, the lack of intensive (i.e., abundant clustering) modifications on the shaft, makes both tibial sides to lack a pattern, with the exception of the lateral and caudal proximal shafts. This moderate clustering there creates the small peninsula between bins 5 and 11 of Fig. 8.

In summary, the humeri, femora and radii-ulnae exhibit strong patterning on how lions modify them after consumption, as reflected in tooth mark distribution on both sides of the same elements. The tibiae display a more variable pattern, which overall is reflected on fewer modifications, especially along the shaft. Given the commonly isolated nature of most marks created along the shaft, these respond more to stochastic processes and reflect higher variability than in the other elements. Exceptions to this observation are found in BSM observed on the tibial crest and proximal caudal-lateral portions of the shaft.

### Multi-element comparison

**Figure 9:**
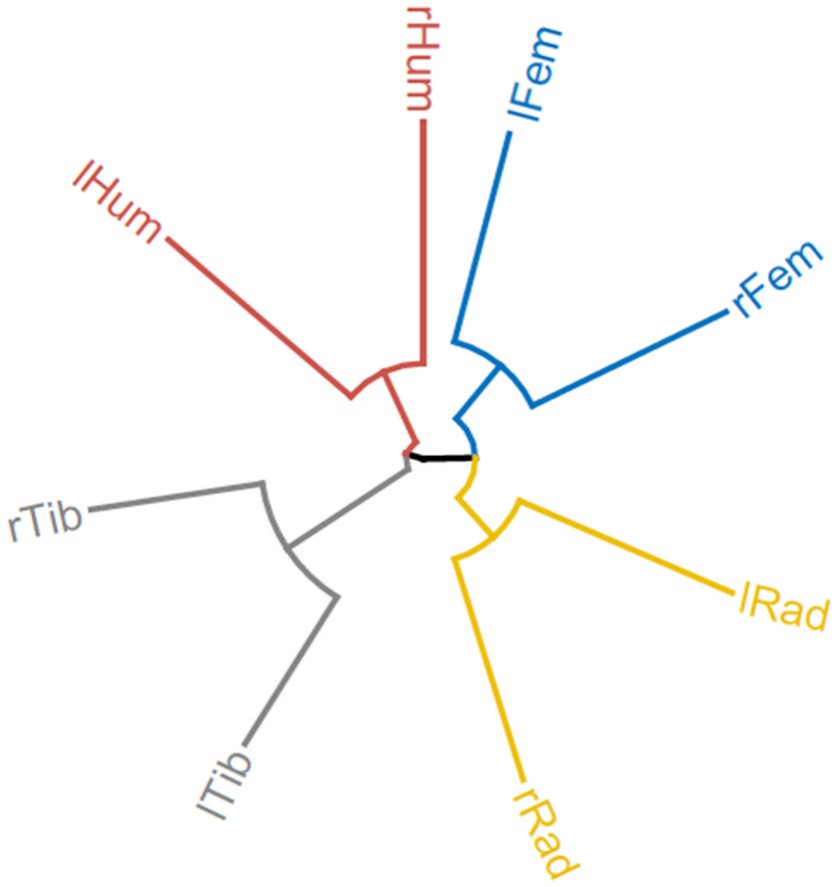
Hierarchical clustering of the selected variables from the second-order functions, intensity, and nearest-neighbour distance. A phylogenetic dendrogram was used. Four groups were identified (different colors) corresponding to each of the four elements analyzed. Key: lHum (left humerus); rHum (right humerus); lFem (left femur); rFem (right femur); lRad (left radius-ulna); rRad (right radius-ulna); lTib (left tibia); rTib (right tibia).

The information contained in the three-dimensional coordinates of the toothmark pattern documented on each of the elements, when approached through the holistic consideration of the mean values of their global interrelation (as documented through the second-order functions), provides identity information (i.e., element-specific identification) for each of the bones analyzed. On a different scale, this could be applied to individual assemblages instead of individual elements as done here. In the comparison among the different elements and their sides, the way marks were distributed in each of their respective point processes (considering their intensity and distances per element) contained sufficient information to differentiate four different clusters corresponding to the four different elements (Table 2, Fig. 9). Within each element set, both sides were contained within the same node. This is of utmost interest, because in the variables used for this analysis, it is the patterns and not the raw coordinates of marks on each element that were used. This enabled the relativization of the actual location of marks on the different long bone elements and only the emergent properties of the mark assemblage in each of them (understood as individual point process) was considered. Thus, multi-element comparison was possible and different bones were successfully differentiated (Fig. 9).

## Discussion

The new Ikhnos 3D software, specifically created for taphonomic and zooarchaeological analysis, constitutes a very useful tool to extract three-dimensional anatomical spatial information of BSM. In the present study, this enabled capturing the subtleties of BSM distribution on carcasses consumed by lions that previous bidimensional methods were not capable of detecting their fullest extent. Through this, comprehensive anatomical patterns of modifications made by lions during carcass consumption were found in all the elements analysed. The adaptation of R code for treating this raw information, mainly through the use of the “spatstat” library, provided the statistical depth that previous GIS approaches to BSM lacked. Here, the patterns documented were derived exclusively through statistical tests and not through visual observation. This makes detection of patterning an analytical procedure and not an inferential one subjected to the subjective appreciation of kernel density maps. The multivariate information thus obtained, through different tests, enabled a comprehensive inter-element approach that enabled the successful classification of all the anatomical parts analysed. Given this new potential gain of information, it is desirable that future taphonomic analyses of BSM make use of this new 3D tool in order to elaborate a comprehensive database and referential models through experimentation. Its potential is not limited to the analysis of BSM, but also to the elaboration of Minimum Number of elements (MNE) and Minimum Number of Individuals (MNI) through the partial overlap of specimens approach, typical of the combination of ArcGIS and the Spatial Analyst module^14^.

Using these latter GIS tools, Parkinson et al.^12^ documented that tooth-marking by captive felids (comprising a combination of tigers and lions) was mostly restricted to long bone ends, with minimal bone fragmentation. On humeri, these authors documented that most damage occurred on the proximal end, near the articular part of the epiphysis and the metadiaphyseal neck, with special emphasis on the posterior and medial aspects. Marks on the distal end were documented mostly on the medial part of the epiphysis. The mid-shaft was virtually devoid of tooth marks. This pattern is mostly like the one described in the present study. The main difference is that Parkinson et al. found more damage on the distal epiphysis than reported here. For the radius-ulna, these authors also documented damage on the proximal and distal portions with minimal modification on the shaft, as documented in our sample. The ulnar olecranon was the most intensively modified portion. Damage reported for the femora is also similar to that described in the present study, with most modifications found on both ends and slightly more tooth marks on the caudal than on the cranial sides of the shaft. For the tibia, the pattern reported by Parkinson et al. is virtually identical to the one we described here, including the clustering of marks on the proximal portion of the caudal shaft on its lateral section. (Although such comparisons must be taken with caution since the bidimensional GIS system used by the Spatial Analyst extension could lead to reproducing the location of the same marks on more than one side, or to subjectively allocate it given the curved nature of the long bone shafts. Here, given the more dynamic nature of the 3D Ikhnos software, the frequencies and actual locations of marks can be better displayed longitudinally on a spatial continuum.) The main difference between Parkison et al.’s study and ours is that in their sample they also documented clusters of tooth marks on the metapodials, whereas those elements in our study were exempt of modification. This is why they were not included in the present analysis. The reasons for these small differences between the two studies may be a combination of factors, including a smaller size carcasses used in Parkinson et al. ’s study, composed of deer (*Odocoileus virginianus*) (Bunn’s^35^ size 2; from 50–100 kg), in contrast with the larger carcasses from our study (Bunn’s size 3: from 100 to 350 kgs). Also, most of the animals used in Parkinson et al.’s work were tigers (*Panthera tigris*) (n = 15) with only 3 African lions (*Panthera leo*) involved. In our study, the number of feeding felids was bigger than in Parkinson et al. ’s study. Most of the experiments used by Parkinson et al. consisted of partial carcasses or even single limbs, although ten complete carcasses were also introduced. The analysis was presented without any possibility of comparing if lions and tigers modified carcasses differently. This is important, since not all felids exhibit the same behavior during feeding: jaguars are more similar to hyenas than to lions in the way that they modify carcasses^10,36^. Tigers are bigger than lions and, therefore, they could inflict more damage on the same carcasses that lions usually feed on. Schaller^37^ describes tiger feeding behavior resulting in that: “The tiger usually eats up its prey so completely that almost nothing remains for scavengers…bones are also swallowed, particularly the soft ones of young animals(p. 300). … unless disturbed, they remain with the carcass until all meat has been devoured” (p 265). This is also what studies of lions in the wild have documented^27,38^, in contrast with biasing partial consumption processes documented with lions in anthropogenically-impacted private reservations^39^, which are the main source of support for interpretations of purported hominin scavenging behaviors^40^. Such interpretations are unsupported by using modern national parks in Africa as proxies for lion carcass consumption behavior^27,38^. Given the overall similarities in Parkinson et al. ’s comprehensive study and ours, it seems that the type of agent (tigers versus lions) does not seem to be an impacting factor and that both agents behave similarly, leaving the same patterning imprints on the carcasses that they consume despite the outlined differences between both studies. It is probably the differences in carcass size that account for the slightly more substantial damage documented by one of the studies.

The present work has refined the patterning in carcass modification by lions documented by previous studies by defining it more comprehensively (quantitatively) at the spatial level and by providing more statistical support. Element-specific patterns have emerged which can be statistically identified. This is relevant, because they provide a lion-specific taphonomic model that reinforces those initially identified by Parkinson et al.^12^ and Organista et al.^26^. Organista el al.’s^26^ larger sample showed (using the element plus bone portion method) that zeugopodials exhibited the lowest rates of tooth marks on shafts, with frequencies that are virtually identical to those reported here.

Highly-debated multiple-agent models that posited that early Pleistocene hominins at sites like FLK Zinj (Olduvai, Tanzania) acquired medium-sized carcasses from felid kills (i.e., lions) have failed to provide any compelling evidence of felid damage on the same elements where hominins left their butchery traces^41^. Hammerstone-broken long bone specimens of proximal and distal stylopodials do not display the typical felid damage documented here. Their surviving humeral articular portions lack the systematic clustering of tooth marks in the metadiaphysis under the articular rim that we document here. The same applies to proximal femoral epiphyseal portions, whose trochanters are not modified felid-style. Distal epiphyses of humeri lack the redundant modification of the medial caudal epicondyle found at lion kills. Distal femoral epiphyses also lack the localized damage imparted by lions and show instead the typical modification imparted by more durophagous carnivores such as hyenas. A single complete femur was presented as proof of felid-hominin interaction^12^, but it was a complete element that contrasted with the bulk of hammerstone-broken specimens that compose the assemblage, including the surviving long bone epiphyses. Given the palimpsest nature of the early Pleistocene record, a pre- or post-deposition of that *Antidorcas* carcass (referred to by Parkinson et al.) by felids independently from hominins seems likely^10^. The definition of the three-dimensional taphonomic signature of this archaeological site as well as those of others should contribute to a better understanding of hominin-other carnivore interactions.

How do the new software and analytical method advance over previous approaches to the study of the anatomical distribution of marks? Previous methods based on kernel map density are basic at the inferential level, since they show intensity areas that are visually (not analytically or statistically) interpreted. Those methods have been applied on templates that represent single elements, on which bilateral right and left sides have not been usually differentiated. If they had, interpretation of patterning could also be subjectively appreciated, since intensity maps may potentially vary on each element side (i.e., one side may show much more intensity than other). Patterning detected through kernel maps is based on absolute intensity location. Patterning detected by the wavelet method presented here does so by comparing mark distribution proportionally, regardless of its intensity. In addition, by using one single side-less element in kernel maps, the resulting “pattern” may be artificial if the true pattern on each side varies. For example, if a carcass lies on one side and either defleshing by carnivores or butchery by humans is more intense on the exposed side, the resulting intensity map may vary substantially between bilateral elements. This variation goes unnoticed when using side-less templates. In such a hypothetical case, the amalgamation of heterogeneous patterns depending on side can generate a kernel map that does not correspond to the real asymmetric distribution of marks. Ikhnos solves this problem by allowing spatially plotting marks by side.

Additionally, on lower-density elements, kernel maps may fail to appreciate clustering or if detected, it provides no indication as to whether it is significantly different from surrounding mark scattering. In this type of low-density point processes, kernel maps tend to over stretch the real clustering areas, creating the false impression that hot spots are bigger than what they actually are^30^. This is due to the fact, as described in the introduction of the present work, that kernel maps are strongly dependent on selected smoothing bandwidths^18–20,30^. The array of methods (and limiting factors) that determine these bandwithds are responsible for highly variable map outcomes when using one method or another^18–20,30,42–44^. Until now, in none of the published works where kernel maps have been adopted, have bandwithds been statistically justified prior to their use. The published maps are, thus, one possibility among several others^42^. This creates uncertainty as to whether any patterning can be objectively derived from such maps. These bandwidth methods are also potentially responsible for the fact that under the same point process, the resulting kernel map could capture or not hot spots or clustering areas, especially when the local intensity varies little compared to the surrounding area^30^. This should not be a problem when local intensity is clearly high compared to the remainder of the immediate bone, but it certainly is an issue when overall intensity is low, which is the case when carcasses are compared at the individual level or in small numbers. One could only speculate as to how different the kernel maps documented previously on carnivore toothmarking^12^ would be if instead of dealing with dozens of carcasses, documentation would have been restricted to just a few. Intrinsic patterning (i.e., the patterning resulting from the ergonomics of carcass defleshing and the channeling of damage imposed by the anatomical distribution of flesh and its attachment to specific areas on bones) can be detected in small samples^45^.Given that kernel maps are strongly dependent on intensity of data points, smaller samples probably would have a major impact on the distribution and extent of the hot zones documented in kernel maps from large-sample studies. Ikhnos and the wavelet method presented here can overcome this problem because patterning is detected regardless of sample size, since it operates on relative mark distribution. It can be applied on single carcasses when comparing sides. When applied on as few as four carcasses, the resulting intrinsic patterning is the same as when documented on many more^45^.

Another advantage of the method presented here is that it can comprehensively analyze all the mark patterning. Kernel maps could potentially be used to detect clusters of marks. However, such clustering may involve only an undetermined fraction of the marks distributed along bones (i.e., only those that cluster). The remaining marks may occur more scattered and thus may go unnoticed in such maps. However, addressing the complete patterning of a mark point process (i.e., clustering and scattering) may be of utmost importance to address behavioral questions. For instance, cut marks have been argued to represent accidents performed during butchery and should therefore reflect stochastic processes. In contrast, butchery ergonomics in combination with the anatomical distribution of muscles should constrict areas where cutmarking will be channeled resulting in intrinsic patterning. The latter process may generate clustering, whereas the former may result in random scattering. Comparing where cut marks are more likely to be clustered or scattered may help understand the global patterning resulting from both randomness and constriction. In this case, determining spatial patterning of scattered marks may be as important as determining clustering. The wavelet method introduced here does so efficiently, by showing simultaneously where clustering and scattered marks occur in a patterned manner on compared elements^42^. In the present work, the element-by-element analysis has shown that scattered tooth marks by lions also show preferential locations on determined bones. This probably indicates that intrinsic patterning of such scattered marks (i.e., those that appear on the high scale of coherence maps) also undergo some degree of channeling during carcass defleshing.

A final improvement on the software and wavelet method is that it is fully analytical. That is, the documentation of the marks at the spatial level are more accurate than in previous kernel models because the template is a 3D bone that is a virtual replica of a real bone. The location of each mark on the curved surface of the element is more accurate than on bidimensional templates. This is followed by each mark having a 3D set of coordinates that enables the whole data set being analysed as a point process and, thus, being interpreted solely on spatial statistical grounds. The analyst does not need to introduce unnecessary (and frequently biasing) subjective interpretations. Coherence graphs and statistical tests define the character of mark associations. As a result, such an analytical approach allows objective comparison among different assemblages through a distance method, which not only shows similarities and differences among assemblages, but also indicate degrees or proximity or separation^42^ (see also Fig. 9). This enables classification of archaeological and paleontological tooth mark datasets according to modern referential datasets.

## Conclusions

In the present study, a three-dimensional taphonomic study of lion modification of long bones from medium-sized carcasses is presented. A pattern of decreasing tooth marking is documented from upper to intermediate long bones (and lower elements, which appear unmodified). Femora and humeri are the most highly modified elements, with special concentration of tooth marks on ends and on or near muscle insertion areas. There are large areas in each of the four bones analyzed, especially around the mid-shaft portion, that contain no tooth marks. Likewise, there are sections within specific bone portions that appear tooth-marked (but more rarely furrowed) around proximal ends which are targets for seeking a lion (felid) taphonomic signature on archaeofaunal assemblages.

The extension of 3D taphonomic modeling to other carnivores opens a window to properly diagnose agency in the anatomical distribution of BSM. It also increases the heuristics for interpretations of archaeological and paleontological assemblages where agent interaction is targeted. In paleoanthropological studies, it promises to provide a testing ground for interpretations of hominins acquiring resources from other carnivores’ kills and for detecting carnivore agent type in the modifications commonly documented in archaeofaunal assemblages.

## Supplementary Information

Supplementary Information

## References

[1] Blumenschine RJ (1995). Percussion marks, tooth marks, and experimental determinations of the timing of hominid and carnivore access to long bones at FLK Zinjanthropus, Olduvai Gorge, Tanzania. J. Hum. Evol..

[2] Pante MC, Blumenschine RJ, Capaldo SD, Scott RS (2012). Validation of bone surface modification models for inferring fossil hominin and carnivore feeding interactions, with reapplication to FLK 22, Olduvai Gorge, Tanzania. J. Hum. Evol..

[3] Domínguez-Rodrigo M, Bunn HT, Yravedra J (2014). A critical re-evaluation of bone surface modification models for inferring fossil hominin and carnivore interactions through a multivariate approach: Application to the FLK Zinj archaeofaunal assemblage (Olduvai Gorge, Tanzania). Quat. Int..

[4] Domínguez-Rodrigo M (2015). Taphonomy in early African archaeological sites: questioning some bone surface modification models for inferring fossil hominin and carnivore feeding interactions. J. Afr. Earth. Sci..

[5] Selvaggio MM, Wilder J (2001). Identifying the involvement of multiple carnivore taxa with archaeological bone assemblages. J. Archaeol. Sci..

[6] Dominguez-Rodrigo M, Piqueras A (2003). The use of tooth pits to identify carnivore taxa in tooth-marked archaeofaunas and their relevance to reconstruct hominid carcass processing behaviours. J. Archaeol. Sci..

[7] Delaney-Rivera C (2009). Pits and pitfalls: taxonomic variability and patterning in tooth mark dimensions. J. Archaeol. Sci..

[8] Andrés M, Gidna AO, Yravedra J, Domínguez-Rodrigo M (2012). A study of dimensional differences of tooth marks (pits and scores) on bones modified by small and large carnivores. Archaeol. Anthropol. Sci..

[9] Arriaza MC (2019). Striped hyenas as bone modifiers in dual human-to-carnivore experimental models. Archaeol. Anthropol. Sci..

[10] Domínguez-Rodrigo M (2015). A new methodological approach to the taphonomic study of paleontological and archaeological faunal assemblages: a preliminary case study from Olduvai Gorge (Tanzania). J. Archaeol. Sci..

[11] Fourvel J-B, Fosse P, Avery G (2015). Spotted, striped or brown? Taphonomic studies at dens of extant hyaenas in eastern and southern Africa. Quat. Int..

[12] Parkinson JA, Plummer T, Hartstone-Rose A (2015). Characterizing felid tooth marking and gross bone damage patterns using GIS image analysis: an experimental feeding study with large felids. J. Hum. Evol..

[13] Madgwick R (2014). What makes bones shiny? Investigating trampling as a cause of bone abrasion. Archaeol. Anthropol. Sci..

[14] Marean CW, Abe Y, Nilssen PJ, Stone EC (2001). Estimating the minimum number of skeletal elements (MNE) in zooarchaeology: a review and a new image-analysis GIS approach. Am. Antiq..

[15] Abe Y, Marean CW, Nilssen PJ, Assefa Z, Stone EC (2002). The analysis of cutmarks on archaeofauna: a review and critique of quantification procedures, and a new image-analysis GIS approach. Am. Antiq..

[16] Parkinson JA, Plummer TW, Bose R (2014). A GIS-based approach to documenting large canid damage to bones. Palaeogeogr. Palaeoclimatol. Palaeoecol..

[17] Stavrova T, Borel A, Daujeard C, Vettese D (2019). A GIS based approach to long bone breakage patterns derived from marrow extraction. PLoS ONE.

[18] Buch-Larsen T, Nielsen JP, Guillén M, Bolancé C (2005). Kernel density estimation for heavy-tailed distributions using the champernowne transformation. Statistics.

[19] Sayah A, Yahia D, Necir A (2011). Champernowne transformation in kernel quantile estimation for heavy-tailed distributions. Afrika Statistika.

[20] Ziane Y, Adjabi S, Zougab N (2015). Adaptive Bayesian bandwidth selection in asymmetric kernel density estimation for nonnegative heavy-tailed data. J. Appl. Stat..

[21] Discamps E (2020). TIPZOO: a touchscreen interface for palaeolithic zooarchaeology towards making data entry and analysis easier, faster, and more reliable. Peer Commun. Archaeol..

[22] Binford, L. R. *Bones: Ancient Men and Modern Myths*. (Academic Press, 1981).

[23] Jiménez-García B, Aznarte J, Abellán N, Baquedano E, Domínguez-Rodrigo M (2020). Deep learning improves taphonomic resolution: high accuracy in differentiating tooth marks made by lions and jaguars. J. R. Soc. Interface.

[24] Yravedra J, Maté-González MÁ, Courtenay LA, González-Aguilera D, Fernández MF (2019). The use of canid tooth marks on bone for the identification of livestock predation. Sci. Rep..

[25] Courtenay LA, Yravedra J, Huguet R (2019). Combining machine learning algorithms and geometric morphometrics: a study of carnivore tooth marks. Palaeogeogr. Palaeoclimatol. Palaeoecol..

[26] Organista E, Pernas-Hernández M, Gidna A, Yravedra J, Domínguez-Rodrigo M (2016). An experimental lion-to-hammerstone model and its relevance to understand hominin-carnivore interactions in the archeological record. J. Archaeol. Sci..

[27] Gidna AO, Kisui B, Mabulla A, Musiba C, Domínguez-Rodrigo M (2014). An ecological neo-taphonomic study of carcass consumption by lions in Tarangire National Park (Tanzania) and its relevance for human evolutionary biology. Quat. Int..

[28] Ripley BD (1976). The second-order analysis of stationary point processes. J. Appl. Probab..

[29] Cressie, N. A. C. *Statistics for Spatial Data*. (Wiley Series in Probability and Statistics New York 1993).

[30] Baddeley A, Rubak E, Turner R (2015). Spatial Point Patterns: Methodology and Applications with R.

[31] Ripley BD (1991). Statistical Inference for Spatial Processes.

[32] Grinsted A, Moore JC, Jevrejeva S (2004). Application of the cross wavelet transform and wavelet coherence to geophysical time series. Nonlinear processes in geophysics. Eur. Geosci. Union (EGU).

[33] Nason G (2008). Wavelet Methods in Statistics with R.

[34] Chavez M, Cazelles B (2019). Detecting dynamic spatial correlation patterns with generalized wavelet coherence and non-stationary surrogate data. Sci. Rep..

[35] Bunn, H. T. Meat-eating and human evolution: studies on the diet and subsistence patterns of Plio-Pleistocene hominids in East Africa. (Ph. D., University of California, Berkeley, 1982).

[36] Rodríguez-Alba JJ, Linares-Matás G, Yravedra J (2019). First assessments of the taphonomic behaviour of jaguar (Panthera onca). Quat. Int..

[37] Schaller GB (2009). The Deer and the Tiger.

[38] Dominguez-Rodrigo M (1999). Flesh availability and bone modifications in carcasses consumed by lions: palaeoecological relevance in hominid foraging patterns. Palaeogeogr. Palaeoclimatol. Palaeoecol..

[39] Domínguez-Rodrigo, M. *Stone Tools and Fossil Bones: Debates in the Archaeology of Human Origins*. (Cambridge University Press, 2012).

[40] Pobiner BL (2020). The zooarchaeology and paleoecology of early hominin scavenging. Evol. Anthropol..

[41] Dominguez-Rodrigo M, Barba R, Egeland CP (2007). Deconstructing Olduvai: A Taphonomic Study of the Bed I Sites.

[42] Peters S, Murphy C, Fan H (2009). Visual bandwidth selection for kernel density maps. Photogrammetrie Fernerkundung Geoinformation.

[43] Shi X (2010). Selection of bandwidth type and adjustment side in kernel density estimation over inhomogeneous backgrounds. Int. J. Geogr. Inf. Sci..

[44] Ruckthongsook W, Tiowari C, Oppong J, Natesan P (2018). Evaluation of threshold selection methods for adaptive kernel density estimation in disease mapping. Int. J. Health Geogr..

[45] Pizarro-Monzo, M., Prendergast, M. E., Gidna, A., Baquedano, E., Mora R., Gónzalez-Aguilera, D., mate-González, M.A., Domínguez-Rodrigo, M. Do human butchery patterns exist? A study of the interaction of randomness and channeling of cut marks on long bones. *J. R. Soc. Interface* (in press) (2020). 10.1098/rsif.2020.0958 PMC7879749 33499767

