# Supplementary material for: A 3D taphonomic model of long bone modification by lions in medium-sized ungulate carcasses
Source: Sci Rep. 2021 Mar 2;11:4944. doi: 10.1038/s41598-021-84246-1 (PMC7925545; doi:10.1038/s41598-021-84246-1)
Supplement: Supplementary file 1 — Supplementary Information [file 41598_2021_84246_MOESM1_ESM.pdf]

SUPPLEMENTARY INFORMATION

A 3D taphonomic model of long bone modification by lions in medium-sized ungulate carcasses.

Manuel Domínguez-Rodrigo[1,2] , Agness Gidna[3], Enrique Baquedano[1], Lucía Cobo-Sánchez[1], Rocio Mora[5], Lloyd A. Courtenay[5], Diego Gonzalez-Aguilera[5], Miguel A. Mate-Gonzalez[5], Diego Prieto-Herraez[5]

[1]Institute of Evolution in Africa (IDEA), Alcalá University, Covarrubias 36, 28010 Madrid, Spain.
[2]Area of Prehistory (Department History and Philosophy), University of Alcalá, 28801 Alcalá de Henares, Spain.
[3]Paleontology Unit, National Museum of Tanzania in Dar es Salaam, Robert Shaban St., P.O. Box 511, Dar es Salaam, Tanzania.
[4]African Wildlife Foundation, P.O. Box 2658, Arusha, Tanzania.
[5]Department of Cartographic and Land Engineering, Higher Polytechnic School of Avila, University of Salamanca, Hornos Caleros 50, 05003 Ávila, Spain

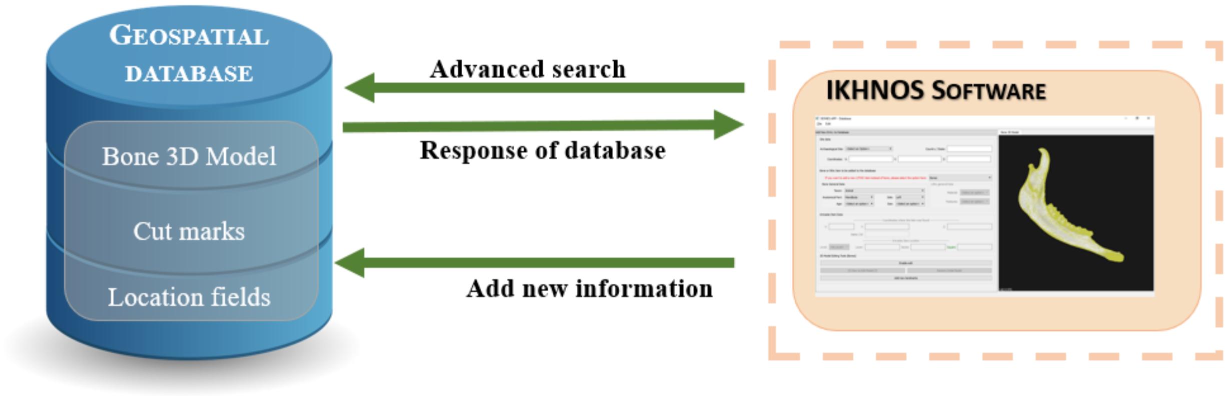

Figure S1. Scheme of connection between the geospatial database and the main application. Database stores new data provided by the user and automatically retrieves the known information.

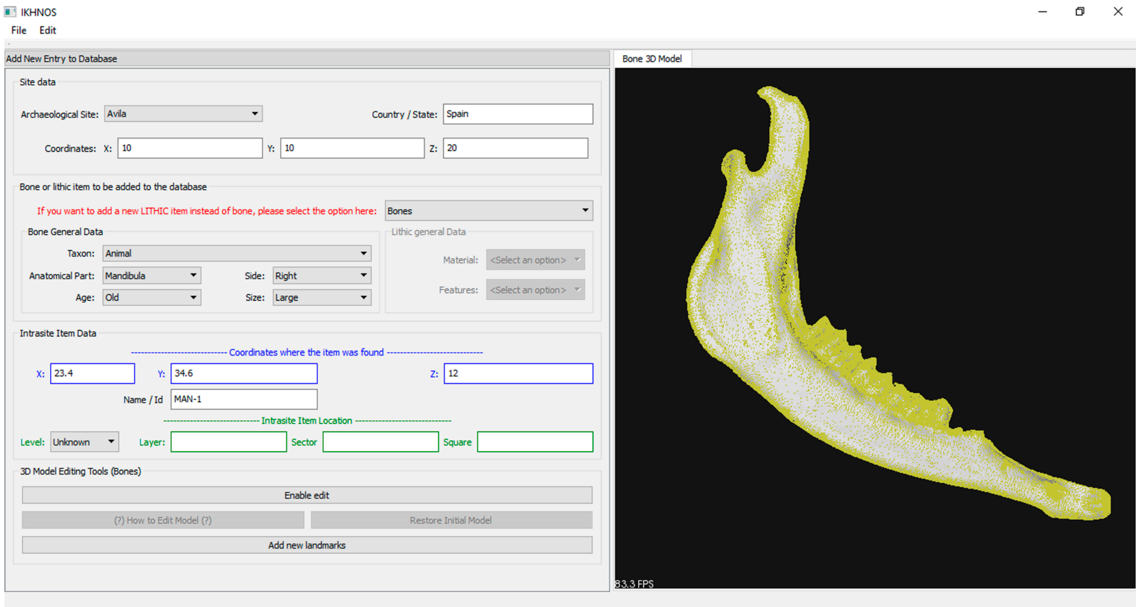

Figure S2. Dashboard of Ikhnos software. (Left) Database information. (Right) 3D viewer of the bone's models.

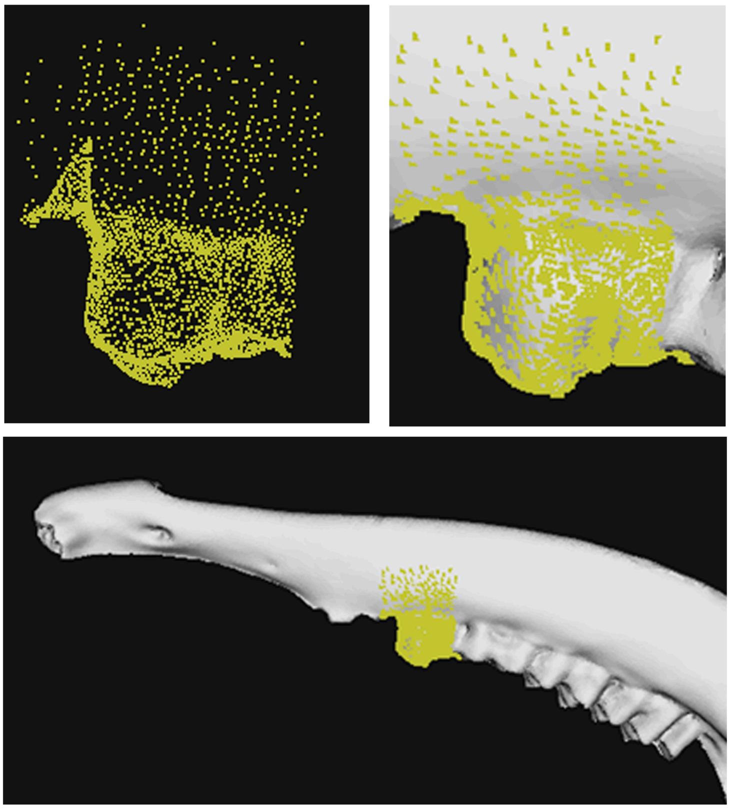

Figure S3. Segmentation of the 3D model to focus on those parts of interest.

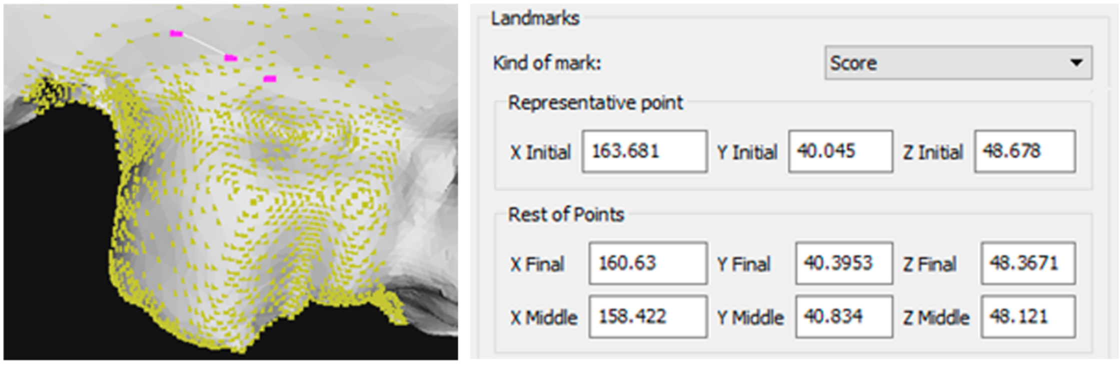

Figure S4. Left: Score landmark selected with three points. Right: Coordinates of these three points.

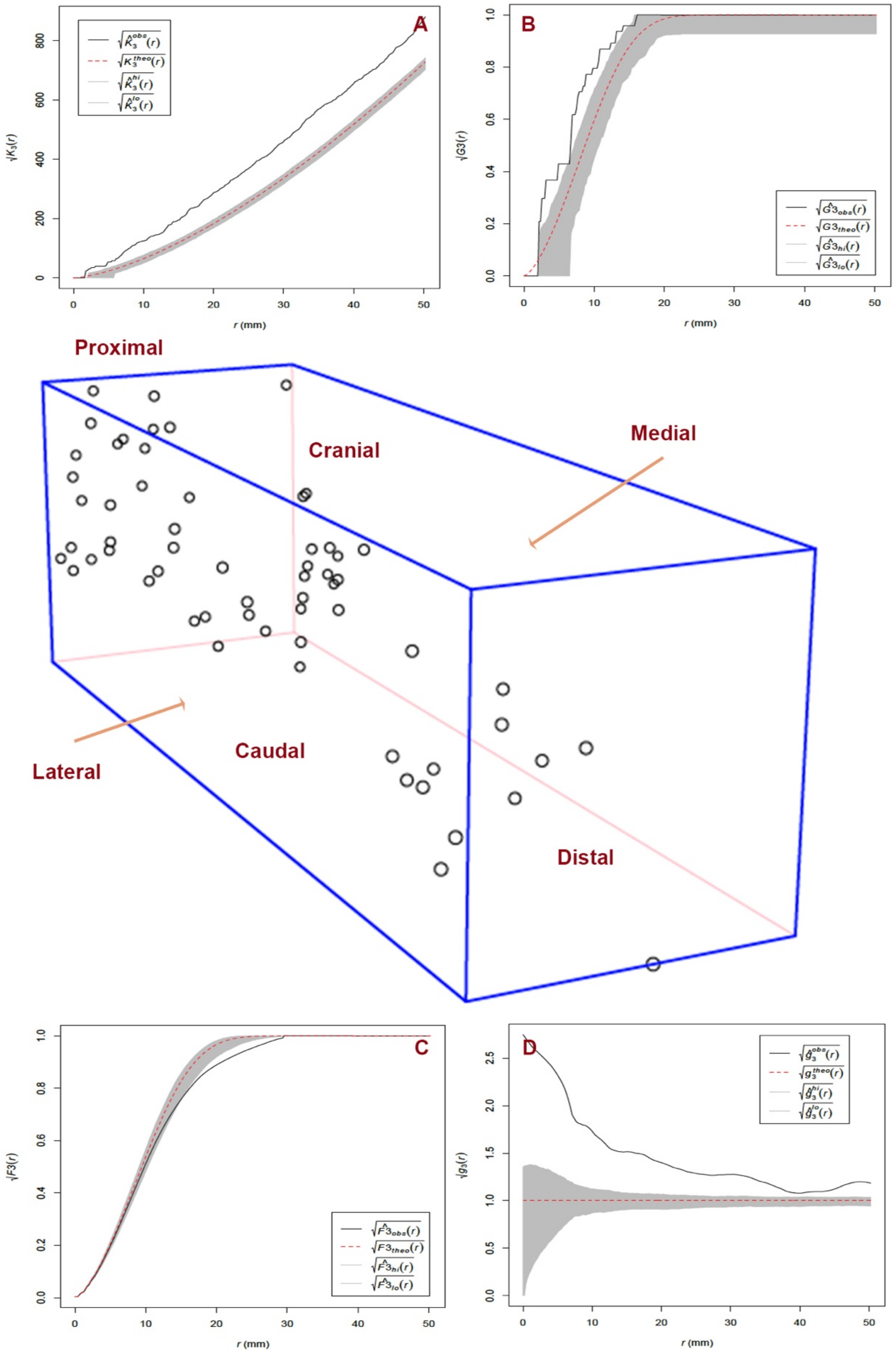

Fig S4. Three-dimensional plot of the distribution of tooth marks on the right humerus. A: K-function plot. B: G near-neighbour function plot. C: F empty space function. D: Pair-correlation function. All functions suggest a mild clustering trend in short distances.

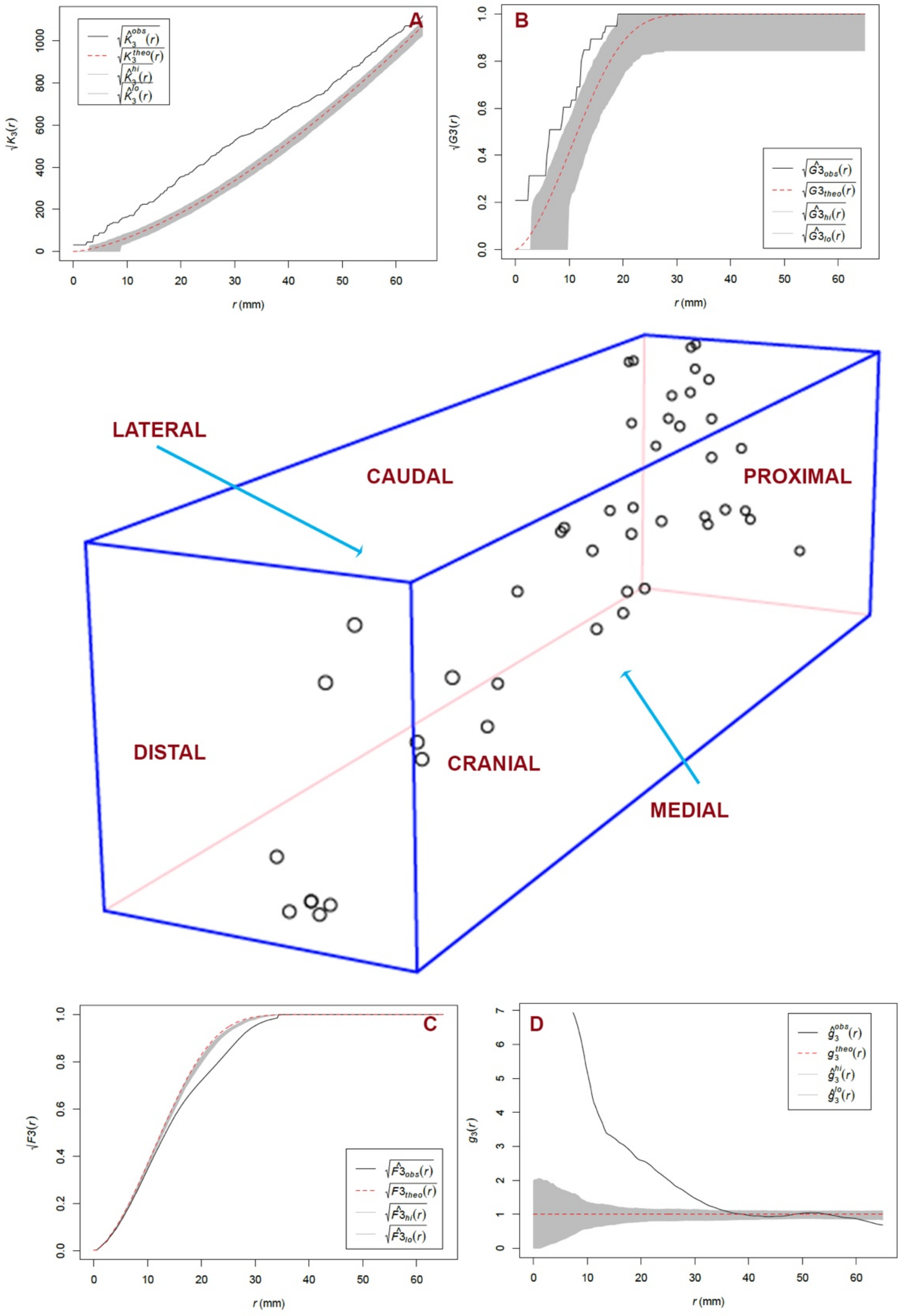

Fig S5. Three-dimensional plot of the distribution of tooth marks on the left femur. A: K-function plot. B: G near-neighbour function plot. C: F empty space function. D: Pair-correlation function. All functions suggest a mild clustering trend in short distances.

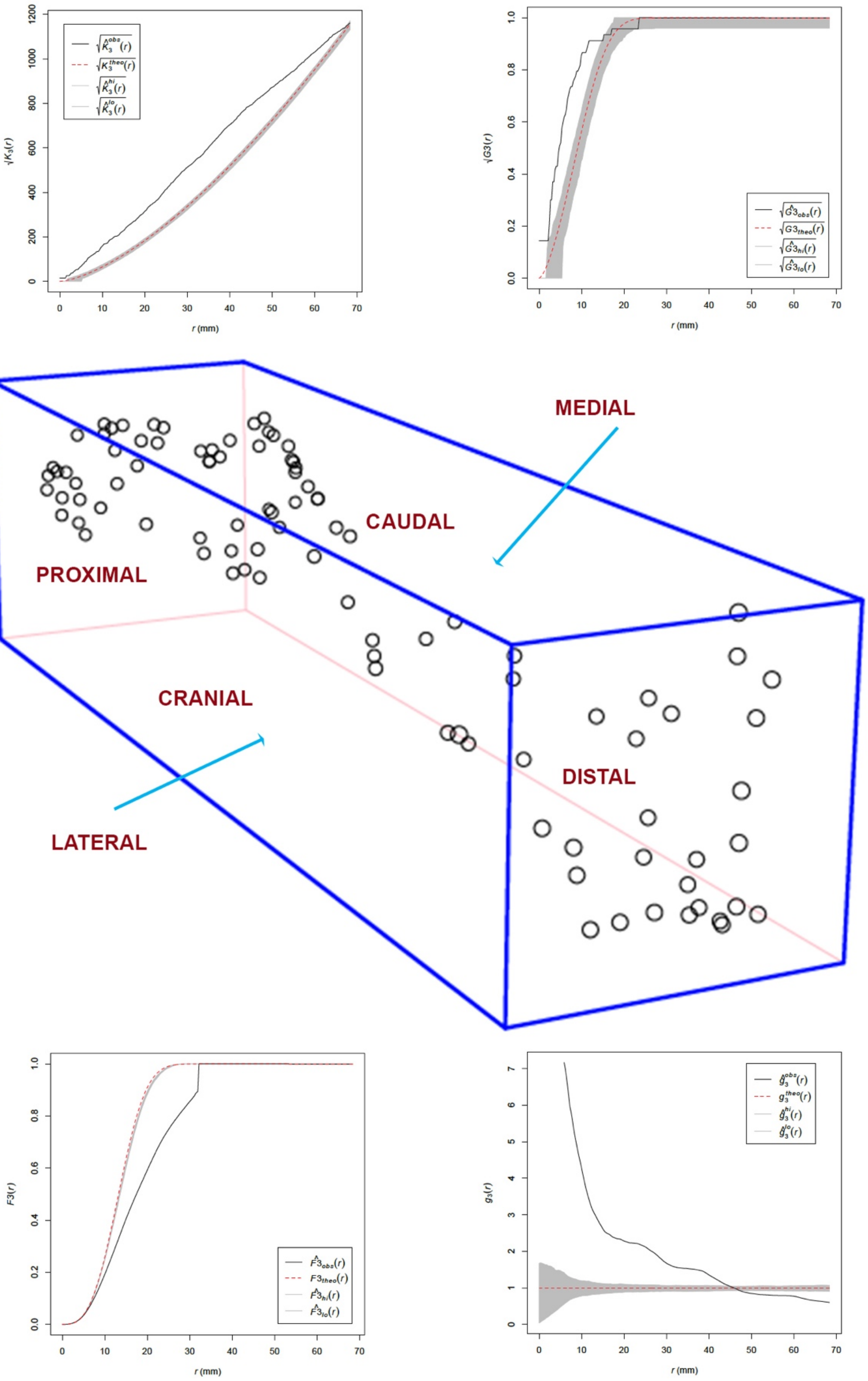

Fig S6. Three-dimensional plot of the distribution of tooth marks on the right femur. A: K-function plot. B: G near-neighbour function plot. C: F empty space function. D: Pair-correlation function. All functions suggest a clustering trend in short distances.

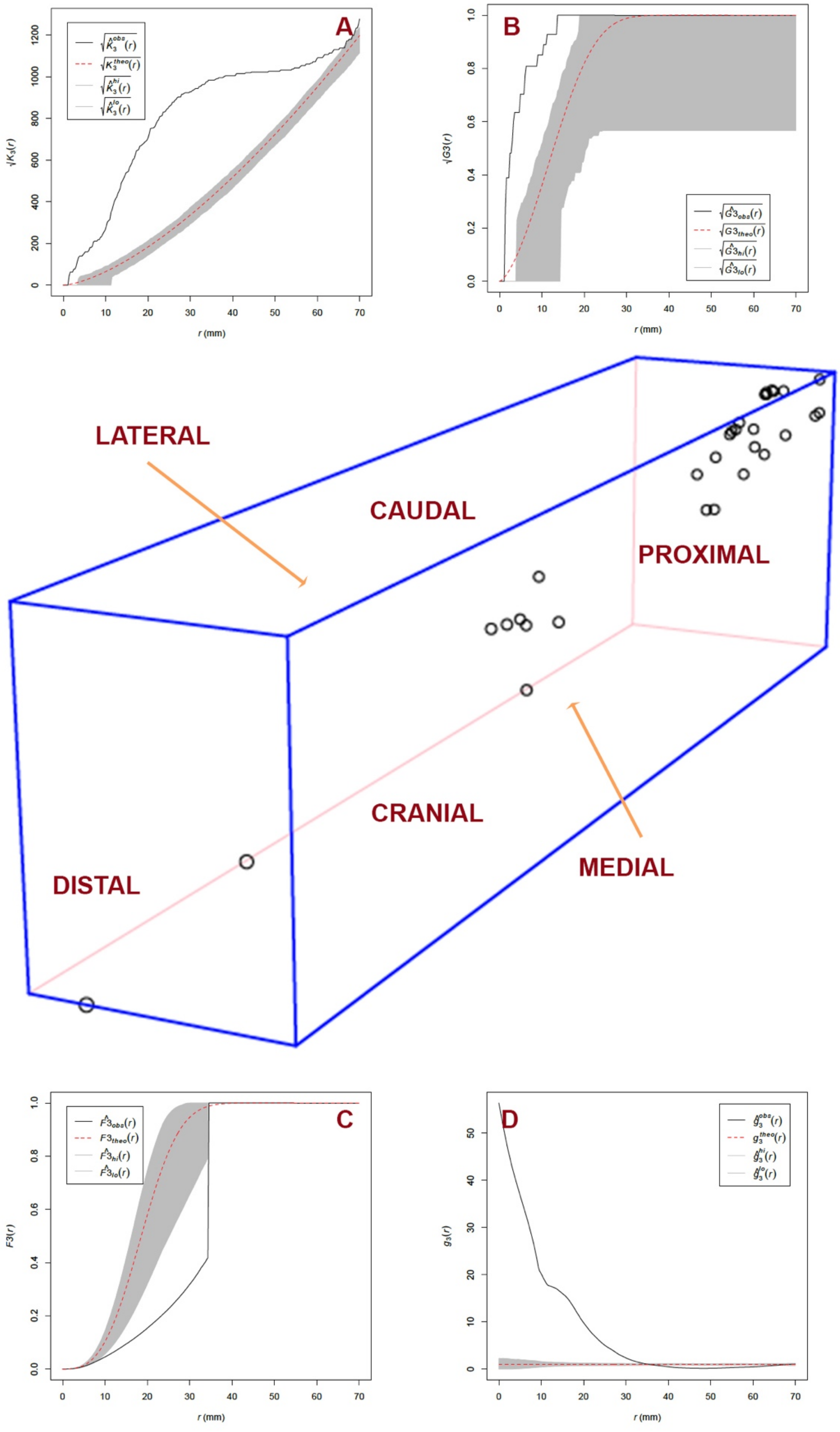

Fig S7. Three-dimensional plot of the distribution of tooth marks on the left radius-ulna. A: K-function plot. B: G near-neighbour function plot. C: F empty space function. D: Pair-correlation function. All functions suggest a marked clustering in short distances.

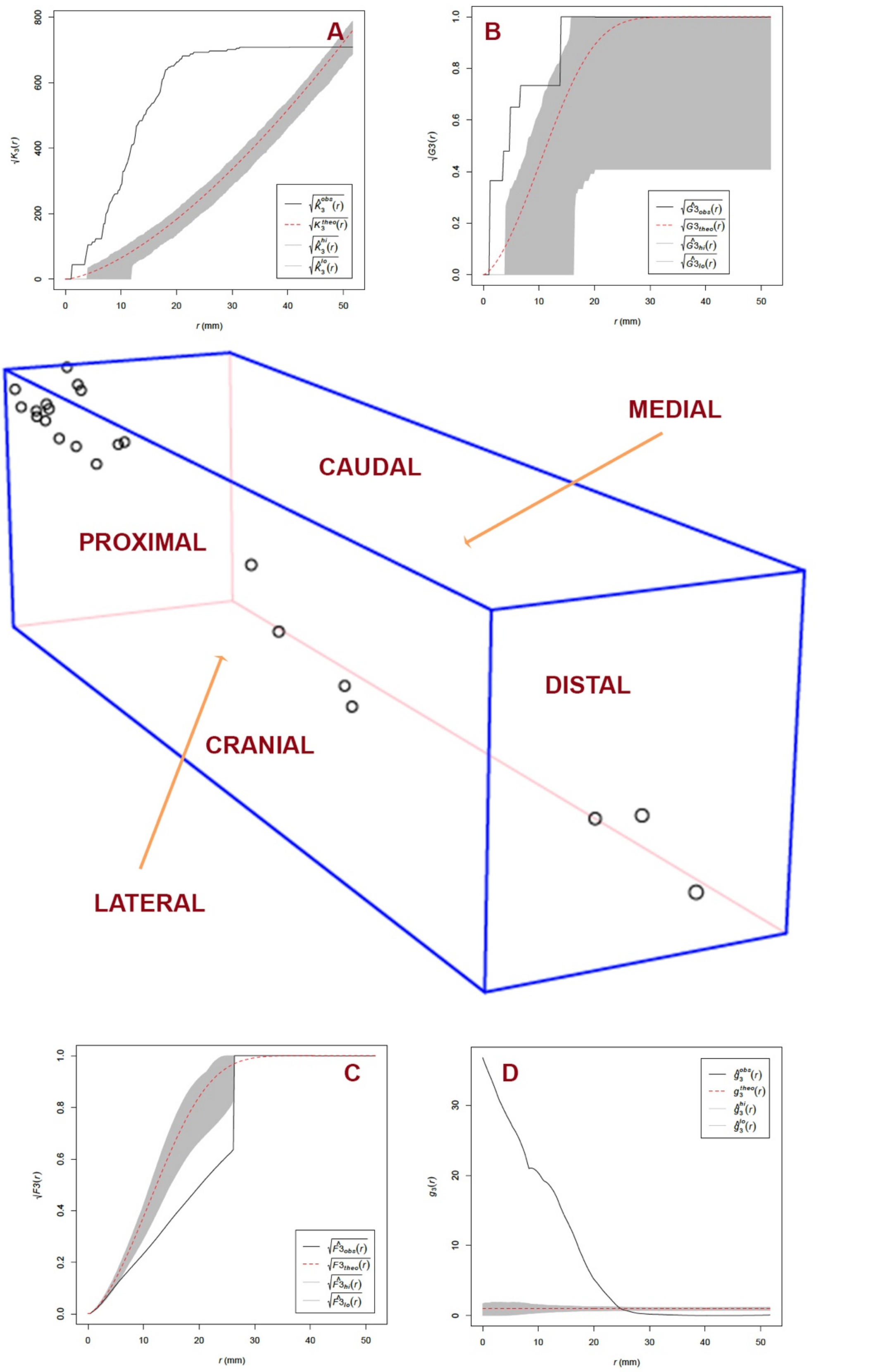

Fig S8. Three-dimensional plot of the distribution of tooth marks on the right radius-ulna. A: K-function plot. B: G near-neighbour function plot. C: F empty space function. D: Pair-correlation function. All functions suggest a marked clustering in short distances.

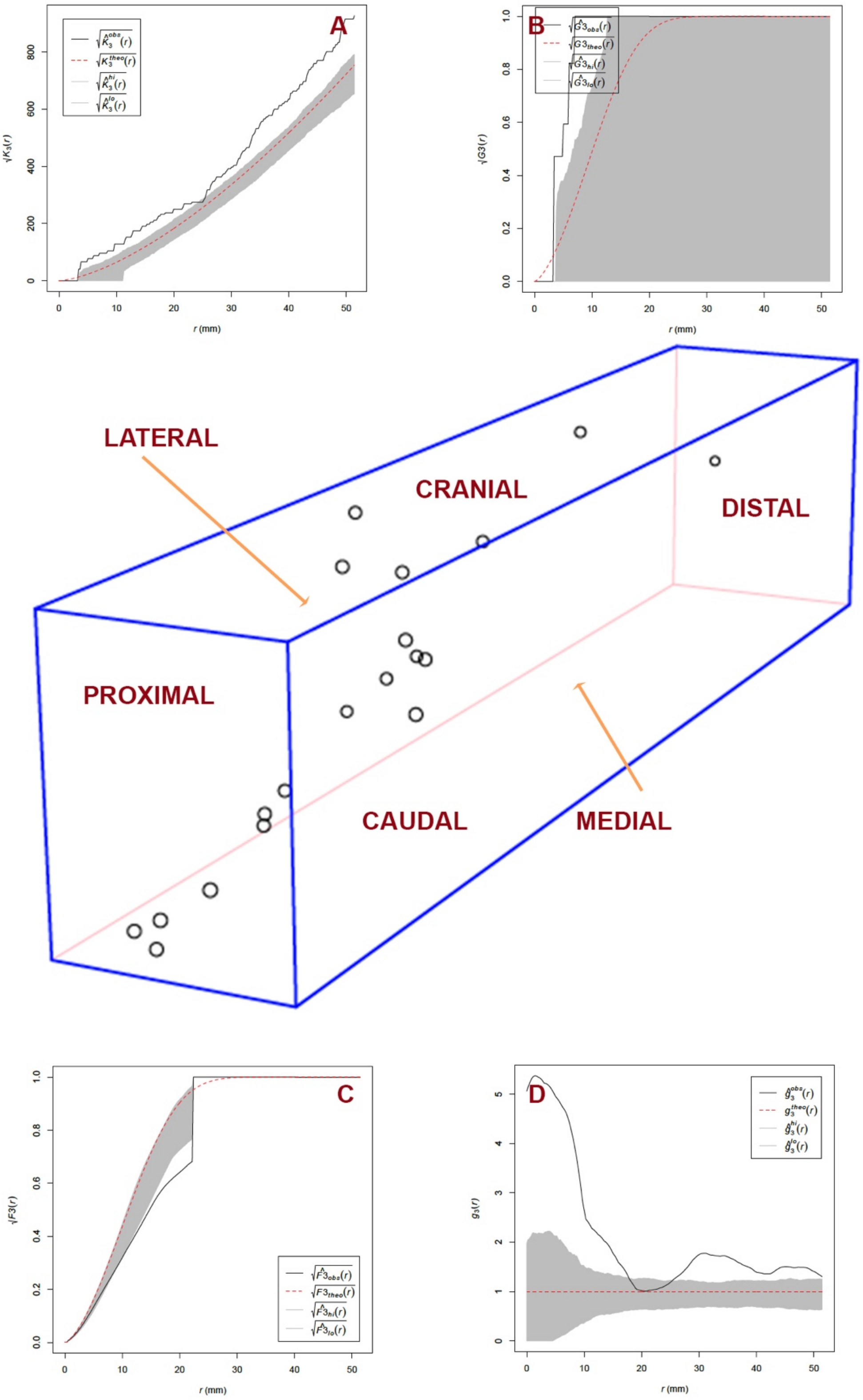

Fig S9. Three-dimensional plot of the distribution of tooth marks on the left tibia. A: K-function plot. B: G near-neighbour function plot. C: F empty space function. D: Pair-correlation function. All functions suggest a minor clustering in short distances, with an overall pattern not far from CSR.

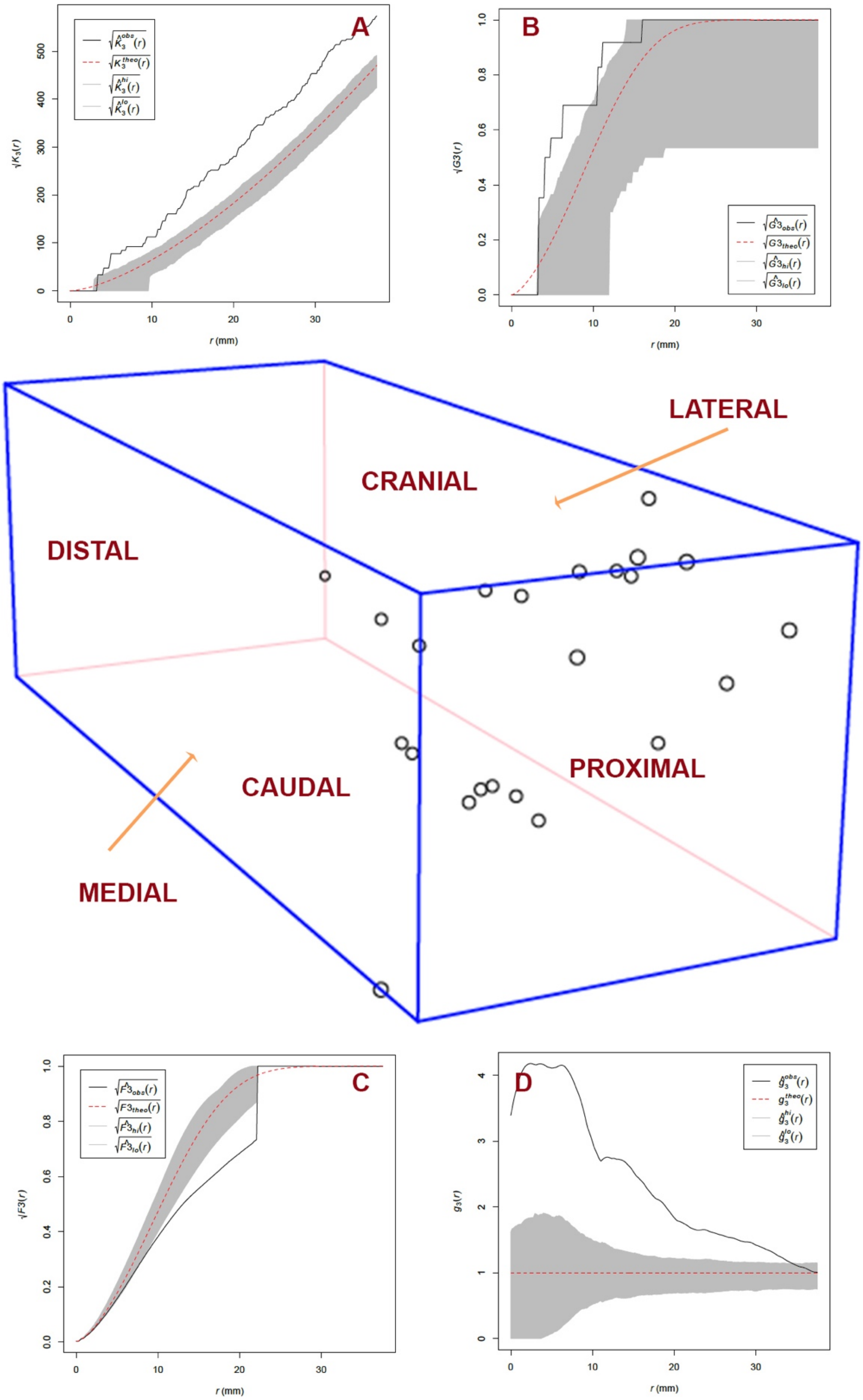

Fig S10. Three-dimensional plot of the distribution of tooth marks on the right tibia. A: K-function plot. B: G near-neighbour function plot. C: F empty space function. D: Pair-correlation function. All functions suggest a minor clustering in short distances, more accentuated than on left tibiae.
